# Consensus statement from the 2025 Delphi panel on cerebral microdialysis in critical care

**DOI:** 10.1186/s13054-026-05993-z

**Published:** 2026-04-13

**Authors:** Adel Helmy, Michael S. Baker, Patrick M. Chen, Aoife Quinn, Ibrahim Jalloh, Louise Roberts, Neeraj Badjatia, Antonio Belli, Martyn G. Boutelle, M. Ross Bullock, Jan Claassen, J. P. Coles, Claire Dahyot-Fizelier, Ari Ercole, Brandon Foreman, Clare Gallagher, Emily J. Gilmore, Arun K. Gupta, Deepak Gupta, Raimund Helbok, Peter Leroux, Sandra Magnoni, Halinder S. Mangat, Niklas Marklund, Anna Teresa Mazzeo, David K. Menon, David W. Nelson, Virginia Newcombe, Mauro Oddo, Kristine O’Phelan, Patrizio Petrone, Maria A. Poca, Ava M. Puccio, Claudia S. Robertson, Elham Rostami, Juan Sahuquillo, Matthew G. Stovell, Anthony J. Strong, Teodor Svedung Wettervik, Eric P. Thelin, Ivan S. Timofeev, Ramon Torné, Alex Valadka, Sara Venturini, Paul Vespa, Chisomo Zimphango, Keri L. H. Carpenter, Jefferson W. Chen, Peter J. Hutchinson

**Affiliations:** 1 Division of Neurosurgery, Department of Clinical Neurosciences, University of Cambridge, Cambridge, UK; 2 Neurology Traumatic Brain Injury & Concussion (NTBIC) Program, Department of Neurology, University of California, Irvine, Irvine, CA USA; 3 Neurosciences Critical Care Unit, Cambridge University Hospitals NHS Foundation Trust, Cambridge, UK; 4 Department of Neurosurgery, Cambridge University Hospitals NHS Foundation Trust, Cambridge, UK; 5 Division of Neurocritical Care & Emergency Neurology, Department of Neurology, Program in Trauma, University of Maryland School of Medicine, Baltimore, MD USA; 6 NIHR Health Protection Research Unit for Emergency Preparedness and Response, University of Birmingham, Birmingham, UK; 7 Neuroscience and Ophthalmology, School of Infection, Inflammation, & Immunology, University of Birmingham, Birmingham, UK; 8 Department of Bioengineering, Imperial College London, London, UK; 9 Department of Neurological Surgery, University of Miami, Miami, FL USA; 10 Division of Critical Care and Hospitalist Neurology, Department of Neurology, Columbia University Irving Medical Center, New York- Presbyterian Hospital, New York, NY USA; 11 Perioperative, Acute, Critical, and Emergency Care (PACE) Section, Department of Medicine, University of Cambridge, Cambridge, UK; 12 Cambridge University Hospitals NHS Foundation Trust, Cambridge, UK; 13 Université de Poitiers, Inserm U1070, PHAR2, Poitiers, France; 14 Service d’Anesthésie-Réanimation et Médecine Péri-Opératoire, CHU de Poitiers, Poitiers, France; 15 Department of Medicine, University of Cambridge, Cambridge, UK; 16 Department of Neurology & Rehabilitation Medicine, University of Cincinnati, Cincinnati, OH USA; 17 Section of Neurosurgery, University of Calgary, Calgary, AB Canada; 18 Department of Clinical Neurosciences, University of Calgary, Calgary, AB Canada; 19 Hotchkiss Brain Institute, University of Calgary, Calgary, AB Canada; 20 Neurocritical Care and Emergency Neurology, Departments of Neurology and Neurosurgery, Yale University School of Medicine, New Haven, CT USA; 21 Department of Anaesthesia, Cambridge University Hospitals NHS Foundation Trust, Cambridge, UK; 22 University of Cambridge, Cambridge, UK; 23 Department of Neurosurgery, JPN Apex Trauma Centre, All India Institute of Medical Sciences, New Delhi, India; 24 Department of Neurology, Kepler University Hospital, Johannes Kepler University Linz, Linz, Austria; 25 Clinical Research Institute for Neuroscience, Johannes Kepler University Linz, Linz, Austria; 26 Bassett Healthcare Network, Cooperstown, NY USA; 27 Anesthesiology and Pain Medicine Service, Department of Medicine, Surgery and Pharmacy, University of Sassari, Sassari, Italy; 28 Department of Neurology, Division of Neurocritical Care, University of Kansas Medical Center, Kansas City, KS USA; 29 Department of Clinical Sciences Lund, Neurosurgery, Lund University, Lund, Sweden; 30 Department of Neurosurgery, Skåne University Hospital, Lund, Sweden; 31 Department of Human Pathology of Adult and Evolutive Age, Division of Anesthesia and Intensive Care, AOU “G. Martino”, University of Messina, Messina, Italy; 32 Department of Perioperative Medicine and Intensive Care, Karolinska University Hospital, Stockholm, Sweden; 33 Department of Physiology and Pharmacology, Section of Anaesthesia and Intensive Care, Karolinska Institutet, Stockholm, Sweden; 34 Innovation and Clinical Research, CHUV-Lausanne University Hospital, University of Lausanne, Lausanne, Switzerland; 35 Neurocritical Care, Department of Neurology, University of Miami Miller School of Medicine, Miami, FL USA; 36 NYU Grossman Long Island School of Medicine, Mineola, NY USA; 37 Department of Neurosurgery, Vall d’Hebron University Hospital, Barcelona, Spain; 38 Neurotraumatology and Neurosurgery Research Unit, Vall d’Hebron Research Institute (VHIR), Vall d’Hebron University Hospital (Vall d’Hebron Barcelona Hospital Campus), Barcelona, Spain; 39 Autonomous University of Barcelona, Bellaterra, Spain; 40 Department of Neurological Surgery, University of Pittsburgh, Pittsburgh, PA USA; 41 Department of Neurosurgery, Baylor College of Medicine, Houston, TX USA; 42 Section of Neurosurgery, Department of Medical Sciences, Uppsala University Hospital, Uppsala, Sweden; 43 Department of Physiology and Pharmacology, Karolinska Institutet, Stockholm, Sweden; 44 Neurosurgery Service, The Walton Centre, Liverpool, UK; 45 Department of Basic and Clinical Sciences, School of Neuroscience, Institute of Psychiatry, Psychology and Neuroscience, King’s College London, London, UK; 46 Department of Medical Sciences, Section of Neurosurgery, Uppsala University, Uppsala, Sweden; 47 Department of Clinical Neuroscience, Karolinska Institutet, Stockholm, Sweden; 48 Medical Unit Neurology, Karolinska University Hospital, Stockholm, Sweden; 49 Department of Neurosurgery, Hospital Clinic de Barcelona, Barcelona, Spain; 50 Department of Neurological Surgery, University of Texas Southwestern Medical Center, Dallas, TX USA; 51 Global Health Research Group on Acquired Brain and Spine Injury, University of Cambridge, Cambridge, UK; 52 David Geffen School of Medicine at UCLA, University of California, Los Angeles, Los Angeles, CA USA; 53 Department of Neurological Surgery, University of California, Irvine, Irvine, CA USA

**Keywords:** Delphi technique, Consensus, Cerebral microdialysis, Acute brain injury, Traumatic brain injury, Subarachnoid hemorrhage, Intracerebral hemorrhage, Neuromonitoring, Multimodal monitoring, Neurocritical care

## Abstract

**Purpose:**

Secondary brain injury is a common cause of poor outcome after trauma, subarachnoid hemorrhage, and intracerebral hemorrhage, and optimizing treatment requires real-time insight into cerebral metabolism. Cerebral microdialysis (CMD) uniquely provides key related information, yet consensus on its use has not been updated since publication of the consensus statement from the 2014 International Microdialysis Forum. We aimed to assess expert consensus on the use of CMD in critical care and provide contemporary guidance to standardize practice and advance clinical implementation.

**Methods:**

We conducted a 3-round modified Delphi study with international experts in CMD and neurocritical care. Consensus was defined as ≥ 75% agreement among non-abstaining respondents, with a minimum of 30 non-abstaining respondents required per statement. Statements not reaching consensus were iteratively revised based on panelist feedback.

**Results:**

Forty of 67 invited experts (60%) from 9 countries participated. Sixty of 62 individual items achieved consensus (97%) across 9 domains: indications and patient selection, technical and procedural considerations, detecting deterioration and secondary injury, metabolic interpretation, treatment algorithms, glucose management, sampling frequency, core reporting items, and barriers to clinical implementation.

**Conclusion:**

This consensus statement provides updated, evidence-informed recommendations for the use of CMD in critical care. The panel reaffirmed many core recommendations from the 2014 consensus while making targeted advances: cautious extension of guidance to intracerebral hemorrhage, comprehensive reporting guidance addressing frequently omitted elements (19 items vs. 6 in 2014), and identification of 10 key barriers to routine clinical adoption.

**Supplementary Information:**

The online version contains supplementary material available at 10.1186/s13054-026-05993-z.

## Introduction

Bedside cerebral microdialysis (CMD) is a monitoring technique that enables continuous sampling of the brain’s extracellular interstitial fluid, providing real-time information on tissue substrate delivery and cerebral metabolism. Since its introduction into critical care over 30 years ago [1], CMD has contributed substantially to the understanding of acute brain injury pathophysiology, particularly in traumatic brain injury (TBI) and subarachnoid hemorrhage (SAH).

In 2014, the International Microdialysis Forum [2] convened in Cambridge, United Kingdom, producing a consensus statement that built upon the original 2004 guidance [3] with substantial advances: enhancing methodological recommendations, introducing core data reporting requirements, defining pathological thresholds for key metabolites, presenting a hierarchy of metabolites’ clinical significance, and offering initial treatment guidance for metabolic derangements. The 2014 consensus statement, which has served as the principal reference framework for CMD in TBI and SAH, acknowledged that there was insufficient evidence at the time to warrant pertinent guidance for other conditions.

In the past decade, clinical experience with CMD has continued to grow not only in TBI and SAH but also intracerebral hemorrhage (ICH). Furthermore, while the 2014 consensus identified core data reporting elements, the increasing complexity of multimodal monitoring and the growing emphasis on standardized research practices have highlighted the need for more comprehensive reporting standards for CMD, especially within the context of prospective trials. In tandem, barriers to the implementation of CMD, which were not addressed in prior consensus statements, have become increasingly apparent and now warrant systematic evaluation.

Catalyzed by the Cerebral Microdialysis Satellite Symposium held at the 2024 International Neurotrauma Society meeting in Cambridge, we aim to update and extend the 2014 consensus through a structured modified Delphi process designed to align international expert practice with the contemporary evidence base. The resulting guidance is intended to supplement, not replace, existing clinical management guidelines. Our objectives were to: reaffirm or refine existing guidance for CMD in TBI and SAH; assess whether expert consensus now supports extending guidance to ICH; and address gaps not covered in the prior consensus, including expanded reporting standards and barriers to clinical implementation.

## Methods

### Study design

We conducted a three-round modified Delphi study (fixed rounds, online administration via Qualtrics) between 25 April and 18 November 2025. The Delphi method is an iterative, anonymous process for achieving consensus among experts, where panelists rate statements, provide feedback, and reconsider their positions in subsequent rounds after reviewing aggregated group responses.

Sixty-seven potential panelists were identified through literature review of CMD publications and consultation with senior researchers in the field. Eligibility criteria included authorship on peer-reviewed CMD publications and/or documented clinical or research experience with CMD in critical care. We sought geographic diversity and representation across clinical and research domains. A writing committee of nine members (AH, MSB, PMC, AQ, IJ, LR, KLHC, JWC, PJH) guided statement development, interpreted results, and revised statements iteratively between rounds, but did not vote and were excluded from consensus calculations in order to preserve independence between content development and voting. The writing committee, which comprised intensivist, nursing, research, and surgical expertise, convened synchronously via video conference and discussed asynchronously throughout the process; all statement development and revision decisions required agreement from the full committee rather than unilateral action by any individual member, mitigating potential bias.

Statement consensus was defined a priori as ≥ 75% agreement among non-abstaining respondents, consistent with established Delphi methodology [4], with a minimum of 30 non-abstaining respondents required per statement.

### Delphi procedure

#### Round 1

Panelists received an initial set of statements across nine domains (see Results), developed from literature review and writing committee input, with statements framed to reflect areas of established evidence as well as areas of uncertainty in current practice. Each statement was accompanied by background context and supporting references. Panelists rated agreement on a five-point Likert scale (Strongly Agree, Agree, Neutral, Disagree, Strongly Disagree) with an option to abstain. For each statement, panelists were invited to provide feedback on how the statement could be modified to increase agreement. Additionally, panelists were invited to suggest entirely new statements to be considered for voting in Round 2. This was the only round in which new statement suggestions were solicited.

#### Round 2

Statements reaching consensus in Round 1 were finalized. Statements not reaching consensus were revised based on panelist feedback and re-presented with a summary of Round 1 results. New statements suggested by panelists in Round 1 were also introduced.

#### Round 3

This process was repeated for statements still not reaching consensus and for new statements introduced in Round 2 that required revision.

Panelists were given at least two weeks to complete each round, with reminders sent as needed.

### Presentation of results

For each consensus statement, we report the final agreement percentage followed by the proportion of agreeing respondents (n) to total non-abstaining respondents (N), presented within brackets. Statements that did not achieve consensus after Round 3 are marked as “No Consensus” with the final agreement percentage. Where relevant, accompanying notes detail responder comments and key considerations from the literature relevant to the statements. Survey instruments and complete round-by-round response data are available as supplementary materials (see Additional file 1).

## Results

### Panelist demographics

Forty international experts (60% of those invited) participated in the Delphi process. Participation rates were 39 (97.5%) in Round 1, 35 (87.5%) in Round 2, and 34 (85%) in Round 3. Two Round 1 responses were received after the analysis cutoff; their qualitative feedback informed statement revisions, but Round 1 quantitative calculations reflect the 37 responses received prior to analysis. One additional panelist joined in Round 2.

Panelists represented nine countries, with the largest representation from the United Kingdom (32.5%) and United States (32.5%), followed by Sweden (12.5%), with the remaining 22.5% comprising experts from Austria, Canada, India, Italy, Spain, and Switzerland. The panel was 70% male and 30% female. The majority were physicians (87.5%) and/or researchers (77.5%).

Median CMD experience was 20 years (IQR 14–24; range 1–30). Thirty-seven panelists (92.5%) reported clinical CMD experience. Among those with clinical experience, 100% had experience with TBI, 75.7% with SAH, and 37.8% with ICH. Thirty-three panelists (82.5%) reported experience with CMD probe placement.

### Overview of consensus results

A total of 62 individual items were evaluated across nine domains: indications and patient selection (3 items), technical and procedural considerations (6), detecting deterioration and secondary injury (2), metabolic interpretation (7), treatment algorithms (7), glucose management (6), sampling frequency (2), core reporting items (19), and barriers to implementation (10). All statements met the minimum threshold of 30 non-abstaining respondents. Sixty items (97%) achieved consensus.

### Domain 1: indications and patient selection

We sought formal inclusion criteria to identify patients most likely to benefit from CMD, maximizing clinical utility while minimizing unnecessary risk from invasive monitoring.

1.1 | *CMD monitoring should be considered for TBI or SAH when there is a concurrent indication for invasive cerebral monitoring (e.g.*,* intracranial pressure) or when access is available at the time of neurosurgical procedure.* [TBI: 91.9% (34/37); SAH: 88.9% (32/36)]

1.1 Notes | CMD for TBI has the strongest evidence base, while use in SAH is practiced at specialized centers for vasospasm and delayed cerebral ischemia detection [5]. Access at the time of neurosurgical procedure refers to the opportunity to place a catheter during, for example, open craniotomy or craniectomy [6].

1.2 | *CMD monitoring should be considered for ICH to monitor the particularly at-risk perihematomal zone in selected cases of severe injury when there is a concurrent indication for invasive cerebral monitoring (e.g.*,* intracranial pressure) or when access is available at the time of neurosurgical procedure.* [85.3% (29/34)]

1.2 Notes | While the evidence base for CMD in ICH is less established than for TBI and SAH, recent studies suggest potential value for monitoring perihematomal metabolism [7–12]. Several panelists emphasized that clinical utility depends heavily on accurate catheter placement in perihematomal tissue, though some comments also supported sampling adjacent normal-appearing brain. Regarding patient selection, one panelist suggested restricting CMD consideration to hematoma volumes > 10 mL. Another noted that centers pursuing early/aggressive surgical intervention (e.g., minimally invasive evacuation or decompressive hemicraniectomy) may use invasive monitoring less frequently, whereas CMD may be more applicable where surgical intervention is less common. Among the 34 non-abstaining respondents, 12 had clinical ICH experience and 22 did not; agreement was similar between groups (83.3% vs. 86.4%, respectively).

1.3 | *Although evidence supporting CMD use in ICH patients is still emerging*,* studies thus far are promising and highlight the importance of continued research in this area.* [88.6% (31/35)]

1.3 Notes | Among the 35 non-abstaining respondents, 12 had clinical ICH experience and 23 did not. Panelists with clinical ICH experience voted more conservatively (75.0% agreement) than those without (95.7%), suggesting that those with direct experience endorsed the statement but with greater caution regarding the strength of the emerging evidence.

Panelist feedback emphasized that clinical considerations across the conditions of TBI, SAH, and ICH include patient age, severity stratification (e.g., comatose patients), and catheter placement location.

### Domain 2: technical and procedural considerations

2.1 | *CMD catheter placement should be performed by an appropriately trained clinician (e.g., neurosurgeon, neurosurgical trainee, or intensivist). A consultant/attending neurosurgeon should have overall responsibility for the procedure and ensure that support is available to manage any complications.* [91.4% (32/35)]

2.1 Notes | While there was consensus on clinician involvement, some panelists emphasized that surgical implementation (e.g., placing a transcranial bolt) should be reserved for those with neurosurgical training (e.g., neurosurgeons, neurosurgical trainees), whereas intensivist procedural involvement could be limited to inserting a CMD catheter through existing cranial access devices.

2.2 | *Dependent on local availability and expertise, CMD catheters can be placed by twist drill hole, transcranial bolt, or during open craniotomy or craniectomy, with each of these procedures being safe and effective.* [97.2% (35/36)]

2.2 Notes | Various CMD catheter placement methods were deemed safe and effective [6, 13, 14], but some panelists remarked that use of a transcranial bolt might be ideal for catheter placement security and replacement throughout monitoring.

2.3 | *The first hour of microdialysate should not be used to make clinical decisions due to the confounders of catheter insertion and pump flushing.* [100% (37/37)]

2.3 Notes | One panelist’s feedback underscored the implication that samples from this early window should also be discarded from the scope of analysis (e.g., retrospective, research), while another noted this period also allows for neuroimaging confirmation of catheter placement. A third highlighted that continuous online CMD systems [15] may stabilize more quickly (20–30 min), potentially enabling earlier clinical insight.

2.4 | *While CMD is an inherently focal monitor, its data may be cautiously interpreted as representative of the hemispheric metabolic state in diffuse injury patterns, provided the catheter tip is placed in brain tissue which does not have a focal injury on neuroimaging.* [94.3% (33/35)]

2.4 Notes | Panelists stressed that heterogenous injury patterns complicate extrapolating data to the whole brain. One panelist highlighted that the default assumption should be that CMD data is locally representative. Evidence on metabolic differences between perilesional and normal-appearing tissue in TBI [16, 17], SAH [5, 18–20], and ICH [8, 12] should be taken into account when generalizing focal CMD results to hemispheric metabolism; however, identifying focal injury remains a neuroimaging challenge [21].

2.5 | *The use of two CMD catheters, which has been implemented safely in published series, can provide a more comprehensive insight than single catheter monitoring in cases with heterogeneous injury patterns.* [82.9% (29/35)]

2.5 Notes | The use of two CMD catheters in heterogeneous injury patterns was supported as an optional adjunct rather than routine practice, consistent with 2014 guidance [2] and more recent evidence [5, 6]. However, some panelists expressed aversion to routine use of two catheters due to the added invasiveness, limited feasibility outside research-active centers, and difficulty justifying this approach as standard of care.

2.6 | *Integration of CMD data with multimodal physiological signals is essential for holistic clinical decision making.* [89.2% (33/37)]

2.6 Notes | Some panelists cautioned that the term ‘essential’ in describing CMD’s role in guiding clinical decisions was an overstatement given the current evidence, with one suggesting ‘valuable’ instead. Nonetheless, panelists agreed that interpreting CMD data in the context of multimodal signals was important, and that CMD could play a helpful and informative role when appropriately integrated with other monitoring data.

### Domain 3: detecting deterioration and secondary injury

A key clinical application of CMD is the detection of secondary insults. Evidence supports CMD’s ability to not only detect but in some cases predict such events.

3.1 | *CMD can provide early indication of impending deterioration and secondary injury*,* such as intracranial hypertension in TBI and symptomatic delayed ischemia in SAH.* [TBI: 89.2% (33/37); SAH: 91.7% (33/36)]

3.1 Notes | In TBI, brain tissue lactate elevations can precede intracranial hypertension episodes, with metabolic failure often occurring before intracranial pressure (ICP) rises [22, 23]. Despite the panel’s consensus on this statement, one panelist suggested that the association may be particularly relevant in diffuse brain injury patterns. Furthermore, another panelist voiced that CMD changes are usually subordinate to ICP and brain tissue oxygenation (PbtO_2_) changes, suggesting that ‘metabolic failure’ is the more appropriate endpoint for CMD monitoring. A panelist noted that predictive associations with secondary insults may be most robust for SAH compared to other conditions. In SAH, there is evidence that ischemic metabolic patterns can precede symptomatic neurological deficits, supporting CMD as a tool for monitoring vasospasm and delayed cerebral ischemia [2, 24–27]. The term “symptomatic delayed ischemia” is used in the statement as presented to panelists, consistent with the 2014 consensus [2]; delayed cerebral ischemia is by definition a clinical (symptomatic) entity. Multiple panelists emphasized that accounting for the location of the CMD catheter is important for reliable deterioration-predictive associations in both TBI and SAH.

3.2 | *CMD is capable of indicating metabolic changes associated with the development of cerebral edema in the perihematomal zone of ICH*,* particularly when the catheter tip is placed in this region.* [77.1% (27/35)]

3.2 Notes | For ICH, the panel advocated for more cautious language, considering the developing evidence base. Panelists noted that metabolic changes may reflect general metabolic vulnerability rather than edema specifically, and precise catheter placement in the perihematomal zone is critical for meaningful interpretation, although, precise placement may prove difficult. Among the 35 non-abstaining respondents, 12 had clinical ICH experience and 23 did not; panelists with clinical ICH experience agreed more strongly (83.3% vs. 73.9%), suggesting that direct clinical experience with ICH supported the statement.

### Domain 4: metabolic interpretation

4.1 | *The most reliable clinical CMD parameters are glucose and the lactate/pyruvate ratio (LPR), which provide more clinically actionable information than monitoring glutamate or glycerol.* [100% (37/37)]

4.1 Notes | The panel unanimously reaffirmed that glucose and LPR are the most clinically actionable CMD parameters, as established in the 2014 consensus [2]. Clinical actionability entails therapeutic interventions available to target these parameters. However, some panelists called into question the ‘reliability’ of these parameters, advising against overstatement of our ability to obtain trustworthy data if there are technical problems with the microdialysis catheter.

4.2 | *For those patients with abnormally high LPR (> 25) detected by CMD despite other multimodal parameters having been normalized in line with locally determined treatment protocols: Ischemia is indicated by concurrently insufficient substrate (e.g., brain glucose < 1.0 mM or pyruvate < 70 µM). Clinical mitochondrial dysfunction is indicated by concurrently sufficient substrate (e.g., brain glucose ≥ 1.0 mM and pyruvate ≥ 70 µM).* [Ischemia: 86.1% (31/36); Mitochondrial dysfunction: 88.9% (32/36)]

4.2 Notes | A central theme in metabolic interpretation is differentiating the etiology of elevated LPR (> 25). Accurate differentiation is required to guide appropriate intervention; for example, emerging therapies such as succinate [28–30] or cyclosporin A [31, 32] specifically target mitochondrial dysfunction. A systematic review established that ‘metabolic crisis’ can be differentiated into ischemia (insufficient substrate) versus mitochondrial dysfunction (adequate substrate but inadequate utilization) [33]. LPR above 25 correlates with worse outcome, while a higher threshold of 40, which is used at some centers and supported by several panelists, does not show stronger association [34]. A glucose threshold of 1.0 mM aligns with neuroglycopenia definitions [30, 35], and 70 µM pyruvate represents the lower bound of multiple groups’ proposals ranging up to 120 µM [36–39]. Some panelists suggested that with the statement’s conditions of substrate sufficiency, the term ‘mitochondrial dysfunction’ may be reductive, preferring the broader term ‘non-ischemic metabolic dysfunction’, which additionally encompasses hyperglycolysis. One panelist stressed that isolated or transient perturbations insufficiently support classification, and that stable trends were crucial; recent work has proposed a framework to differentiate sustained metabolic derangements from transient fluctuations [40]. Furthermore, multiple panelists emphasized that CMD data should be integrated with other modalities for definitive diagnosis, particularly brain oxygenation given its ability to aid in identification of hypoxia-driven LPR elevations [41], though CMD’s focal nature may limit direct correlation with other regional monitors.

4.3 | *Absolute lactate and pyruvate concentrations, alongside glucose, should be considered when interpreting heightened LPR, as this can indicate if the abnormality relates to a failure of metabolic substrate delivery.* [94.6% (35/37)]

4.3 Notes | The panel emphasized that practical LPR interpretation requires examining concurrent CMD analyte concentrations; lactate [42], pyruvate [36–39], and glucose [30, 35, 40] concentrations help differentiate ischemic from non-ischemic energy failure.

4.4 | *The interpretation of an elevated LPR should consider the trend over time and the duration of the elevation, as an isolated value may be insufficient to change clinical management.* [97.1% (34/35)]

4.4 Notes | This statement supports the importance of temporal patterns: trends and duration are more reliable than isolated values, which can be misleading due to transient events or artifacts. However, some panelists highlighted that transient disturbances as indicated by CMD (e.g., corresponding to ischemia or an episode of arterial hypotension leading to metabolic failure) may still hold meaningful pathophysiological weight if concordant with the clinical and monitoring picture.

4.5 | *Elevated cerebral glycerol is an indicator of cell death and damage from secondary injury after TBI.* [85.3% (29/34)]

4.5 Notes | A few panelists described CMD glycerol as nonspecific and frequently erratic. While confounders such as catheter insertion and systemic origins (e.g., glycerol-containing drugs) were noted as potential concerns, evidence suggests that systemic administration has minimal impact on cerebral glycerol levels [43]. Some panelists also noted that glycerol may be a delayed marker of damage that has already occurred, with swift rises in glycerol holding more revealing information about tissue injury or infarction, whereas glutamate may indicate ongoing sources of cellular damage.

4.6 | *An increase in cerebral glutamate is indicative of secondary injury, such as from excitotoxicity, after TBI.* [79.4% (27/34)]

4.6 Notes | It was suggested that increases in CMD glutamate could indicate active (one panelist suggested potentially reversible) episodes of excitotoxic secondary injury, perhaps corresponding to spreading depolarizations [44], although glutamate, like glycerol, lacks inherent specificity.

4.7 | *Increased cerebral glutamate is associated with poor clinical outcome.* [**No Consensus**: 73.5% (25/34)]

4.7 Notes | Panelists speculated that a lack of consensus on the correlation of CMD glutamate with outcome may be due to the duration of increased glutamate not being taken into account, and that reversible causes of glutamate increase (e.g., seizures) and associated injury may be treated and thus obfuscate association with outcome.

### Domain 5: treatment algorithms

5.1 | *In a prioritized approach to treating deranged metabolism with the intention of lowering the LPR, the following general sequence of priorities is suggested, while acknowledging that these parameters are often managed simultaneously depending on the clinical context: (1) ICP should be addressed with priority (target ≤ 20 mmHg), (2) then PbtO₂ (target ≥ 15 mmHg), (3) and finally, brain glucose should be monitored, with investigation and management of systemic causes for levels below 1.0 mM. (4) Further interventions (e.g., targeting cerebral perfusion pressure [CPP]) should be considered for refractory metabolic derangement as indicated by a persistently increased* LPR (> 25). [(1–3): 94.1% (32/34); (4): 79.4% (27/34)].

**Fig. 1:**
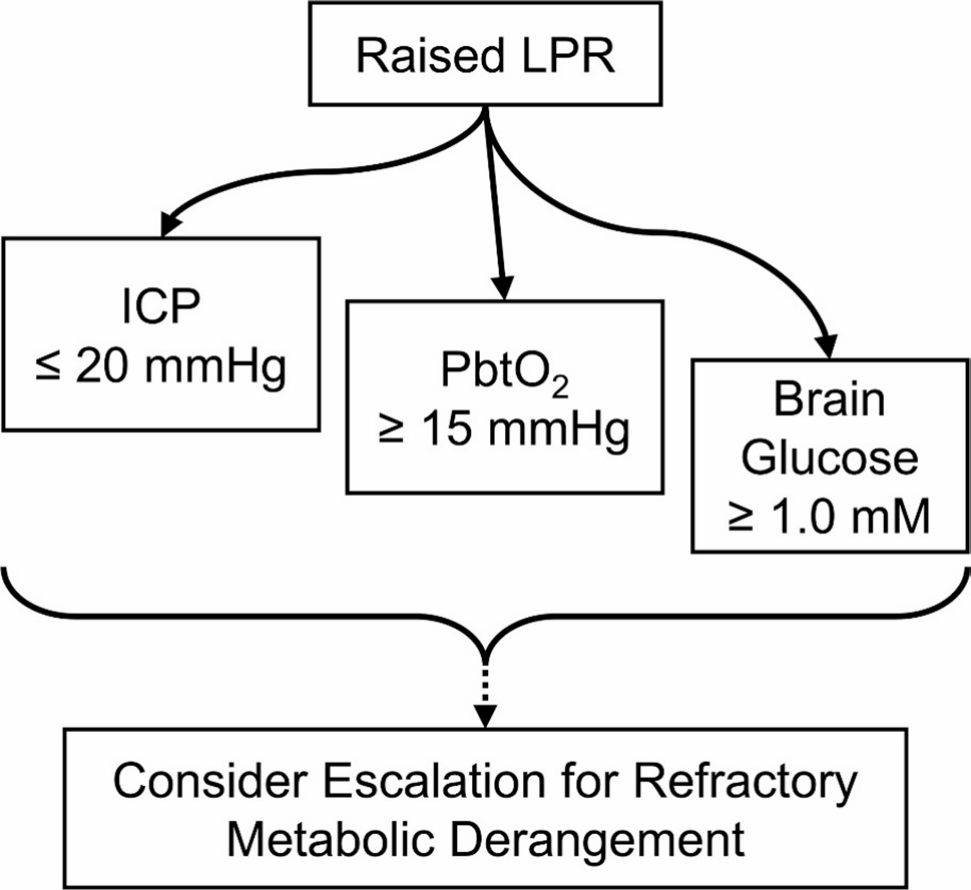
Targets for treating deranged metabolism as indicated by raised LPR. Arrows from “Raised LPR” branch simultaneously to three targets (ICP, PbtO₂, brain glucose), reflecting their concurrent management in clinical practice, often in conjunction with CPP optimization. Staggered positioning indicates the relative priorities voted on by panelists, though all three are typically addressed in parallel. Safe serum glucose ranges (< 10 mM) should be maintained even when treating neuroglycopenia. If LPR remains persistently elevated (> 25) despite optimization of these targets, further escalation should be considered. **LPR**, lactate/pyruvate ratio; **ICP**, intracranial pressure; **PbtO₂**, brain tissue oxygenation; **CPP**, cerebral perfusion pressure

5.1 Notes | The panel supported a framework for treating metabolic derangement (see Fig. 1), building on prior metabolic support strategies [30, 35, 40]. However, panelist feedback heavily stressed that a strict stepwise approach lacks practicality, as these multimodal parameters are often managed simultaneously (e.g., CPP optimization in parallel with ICP management, sometimes with the endpoint of PbtO₂); moreover, some centers may not be equipped with every modality in the sequence. Thus, the suggested sequence is meant to serve as a conditionally modifiable guide rather than a strict, comprehensive rule. Several panelists advocated for CPP optimization above targeting PbtO₂, noting that fraction of inspired oxygen manipulation may be futile when oxygen delivery is compromised by inadequate perfusion. Regarding thresholds, an evidence-based PbtO₂ target of 15 mmHg was suggested [45, 46], though thresholds should be interpreted as guidance rather than absolute; one panelist noted that dose (particularly for ICP, for which guidelines may alternatively suggest 22 mmHg) may be more important than isolated numerical values. One panelist also warned of hyperperfusion injury, which may elevate LPR despite normal glucose and ICP. Multiple panelists noted that both low glucose and elevated LPR can independently indicate metabolic insufficiency. Relating to brain glucose (point 3), a panelist emphasized that active efforts to increase cerebral blood flow (CBF) or CMD glucose should accompany monitoring of neuroglycopenia, as monitoring alone is passive; however, some panelists recognized that targeting brain glucose, while theoretically desirable, is not yet standard practice or strongly evidenced, and that safe serum glucose ranges (e.g., below 180 mg/dL or 10 mM) should be maintained. A panelist suggested that EEG findings may provide valuable context regarding seizures, consistent with another panelist’s suggestion of incorporating glutamate into guidance. Interventions mentioned by panelists include sedation for reducing metabolic demand and, if ICP is controlled, cautious adjustment of arterial PCO₂ for improving perfusion. Furthermore, one panelist advocated for individualization of treatment using cerebral autoregulatory indices alongside accounting for correlations with ETCO₂. Lastly, several panelists underscored the relevance of catheter placement (e.g., lesion-relative location) in interpreting CMD data and identifying deranged metabolism.

5.2 | *Although a CPP of 60–70 mmHg is recommended within Brain Trauma Foundation Guidelines*,* metabolic state as indicated by CMD has the potential to inform individualization of CPP targets.* [86.5% (32/37)]

5.2 Notes | The panel agreed that CMD could potentially inform individualization of CPP targets (Brain Trauma Foundation recommends 60–70 mmHg [47]), provided it complements rather than replaces optimization guided by cerebral autoregulation [48] and PbtO₂. Additionally, panelists emphasized that CMD’s focality must be considered when using local metabolic data to guide global CPP targets.

5.3 | *Disturbance of the brain’s metabolic state as indicated by CMD can guide de-escalation of therapy because it demonstrates a remaining vulnerability to physiological perturbations.* [82.9% (29/35)]

5.3 Notes | Panelists stressed that CMD values should be interpreted as additional information to other clinical and neurological parameters (e.g., ICP, PbtO₂, CBF, clinical and radiological examination) rather than in isolation when guiding de-escalation. One panelist emphasized that it is essential to interpret CMD findings in the context of catheter location in considering the generalizability of focal CMD indications to global brain metabolic state and vulnerability. Another panelist emphasized that CMD guidance may entail indication for a closer review of the patient, consideration of repeat imaging, or maintaining tight physiological control. A caveat was proposed that refractorily (i.e., non-dynamically) abnormal CMD findings may be less relevant for de-escalation guidance, whereas dynamically abnormal CMD findings responsive to treatment are more relevant. Similar considerations apply to statement 5.4.

5.4 | *Worsening CMD values may indicate tissue vulnerability and can help inform the decision to defer*,* modify*,* or abort a neurological wake-up test or a trial of sedation lightening.* [88.2% (30/34)]

5.4 Notes | This statement was introduced in light of the association between deranged metabolism and wake-up test failure in severely brain-injured patients [49]. Some panelists observed that the low temporal resolution of CMD (typically hourly sampling) limits its utility for real-time decisions during brief neurological wake-up tests, with its value instead in identifying pre-existing metabolic instability that may warrant deferring a test or in monitoring longer trials of sedation lightening. Some panelists noted insufficient evidence, while one panelist questioned the practice of wake-up tests altogether in patients requiring invasive cranial monitoring.

5.5 | *When taken in a wider context including systemic physiology and cerebral oxygenation*,* raised LPR (> 25) can contribute to a decision to perform red blood cell transfusion (RBCT) in order to improve cerebral oxygenation.* [85.3% (29/34)]

5.5 Notes | While recent randomized trials generally favor liberal transfusion thresholds (hemoglobin 9–10 g/dL) in acute brain injury [50–53], the panel endorsed LPR as one contributing factor to further individualize RBCT decisions in a broader clinical context (e.g., ICP, CPP, PbtO2). A few panelists suggested PbtO2 < 20 mmHg [54] for RBCT suitability. Some panelists remarked that LPR may hold particular utility when hemoglobin is in a borderline range, but that caution and close clinical review should be undertaken because RBCT is not a benign treatment. An LPR of 25 represents the floor of potential ‘raised LPR’ thresholds (e.g., some centers use an LPR threshold of 40 instead). Evidence supports the relationship between anemia and metabolic distress [55, 56], though a subsequent study found RBCT was not associated with metabolic benefits [57].

5.6 | *In TBI patients with a CMD pattern suggestive of ischemia (e.g.*,* LPR > 25 and pyruvate < 70 µM)*,* the initial response should be to evaluate and optimize cerebral perfusion pressure*,* brain tissue oxygenation*,* cerebral autoregulation*,* and brain glucose levels. If the ischemic pattern persists despite these measures*,* a short (e.g.*,* up to 2 h) trial of normobaric hyperoxia (NBO) may be considered as a diagnostic challenge to assess the responsiveness of the metabolic derangement.* [76.5% (26/34)]

5.6 Notes | Focus was limited to TBI given insufficient evidence for SAH or ICH guidance [58]. There may be particular CMD-indicated benefit of NBO when pyruvate and lactate are < 120 µM [38] and > 3.5 mM [59, 60] respectively. With respect to the initial optimization steps, one panelist noted that cerebral autoregulation is a physiological state rather than a directly modifiable target, clarifying that the practical implication is to ensure adequate CBF. Additionally, CPP optimization may affect brain glucose levels. One panelist added the caveat that the probe should not be placed in a contusion. For NBO itself, although studies have shown measurable metabolic benefit, the duration of benefit remains unclear [59, 61], and uncertainty was raised about how such benefit would manifest clinically. The panel emphasized that any hyperoxia trial should weigh risks and benefits for the individual patient.

5.7 | *In patients undergoing ICH resection*,* CMD may be considered as an adjunct to standard monitoring to assess the metabolic response to surgery. With the catheter tip placed in the perihematomal zone*,* worsening or non-recovering metabolic derangements despite resection may signal an evolving secondary injury*,* prompting further investigation to identify potential causes.* [**No Consensus**: 60.6% (20/33)]

5.7 Notes | Panelists desired more evidence, although there are promising single-center results [11, 12]. Multiple panelists noted that accurate perihematomal catheter placement is technically challenging, and distinguishing metabolic derangements from expected post-surgical changes remains difficult. One panelist noted that ‘evacuation’ is the appropriate terminology for hematoma removal; this was identified in feedback from the pre-specified final round, so there was no opportunity to revise the statement. Among the 33 non-abstaining respondents, 11 had clinical ICH experience and 22 did not; panelists with clinical ICH experience agreed more (72.7% vs. 54.5%), though neither group reached the 75% consensus threshold.

### Domain 6: glucose management

6.1 | *In the traumatically injured brain, interstitial fluid glucose measured by microdialysis in tissue presumed to be uninjured has a positive correlation with plasma glucose. Consequently, brain glucose can be modulated by altering plasma glucose levels (e.g., via insulin or glucose). However, the predictability of this response should be determined empirically, as the relationship is influenced by factors such as catheter tip location, local CBF, and metabolic rate.* [88.6% (31/35)]

6.1 Notes | The 2014 consensus [2] acknowledged a positive linear relationship between peripheral and brain glucose in non-injured TBI tissue [62, 63], though this relationship is modulated by local factors, making interventional responses variable [64]. Glucose utilization may be markedly higher in pericontusional and more vulnerable brain regions [65]. One panelist cautioned that low CMD glucose may reflect local flow-demand mismatch rather than systemic insufficiency, and that raising plasma glucose globally in response to a focal signal risks hyperglycemia.

6.2 | *Systemic (plasma) glucose levels should be considered when interpreting cerebral microdialysis glucose data.* [97.1% (33/34)]

6.2 Notes | While reduced brain/serum glucose ratios have been shown to predict metabolic distress and mortality [66], the absolute cerebral glucose value remains clinically critical.

6.3 | *Administration of intravenous dextrose may be used to raise plasma glucose up to 10 mM (180 mg/dL) in an effort to resolve neuroglycopenia.* [75.8% (25/33)]

6.3 Notes | The plasma glucose ceiling of 10 mM (180 mg/dL) aligns with conventional glycemic control, which is less associated with neuroglycopenia than tight control [67–71].

6.4 | *Despite the association of increased cerebral glutamate with brain glucose > 5.0 mM, there is insufficient evidence to define a critical upper limit for brain glucose.* [91.2% (31/34)]

6.4 Notes | Discussion of optimal brain glucose range pervades the literature [72], but the relationship between LPR and glucose is only well-defined below 1.0 mM [73], and the panel agreed there is insufficient evidence to define a critical upper limit. Although increased cerebral glutamate (accepted as a marker of secondary injury, see Statement 4.6) is associated with brain glucose above 5.0 mM [74], this association alone does not warrant avoidance of concentrations above that threshold.

6.5 | *Strategies to treat neuroglycopenia should include optimizing enteral nutrition.* [82.4% (28/34)]

6.5 Notes | Optimizing enteral nutrition [75] was supported as a complementary strategy, acknowledging that its effect on brain glucose is slower than intravenous administration.

6.6 | *The administration of alternative energy substrates, such as lactate or ketone bodies, to manage neuroglycopenia is an investigational strategy that requires further study before it can be recommended for clinical use.* [87.9% (29/33)]

6.6 Notes | Alternative energy substrates such as lactate, ketone bodies, and, as one panelist suggested, succinate [28–30], were characterized as investigational, requiring further study before formal clinical recommendation.

### Domain 7: sampling frequency

7.1 | *Hourly CMD sampling is sufficient to add clinical benefit.* [81.1% (30/37)]

7.1 Notes | Several panelists supported the statement but noted that hourly sampling is suboptimal for multimodal triangulation of pathophysiology or detecting rapid metabolic changes (e.g., during acute deterioration); some panelists suggested that 20–30-minute sampling intervals may offer a practical yet more demanding compromise for current manual sampling workflows.

7.2 | *Higher frequency sampling (sub-hourly) or continuous monitoring may increase the utility of CMD as online monitoring systems are developed.* [81.1% (30/37)]

7.2 Notes | Responses highlighted shared sentiment regarding manual versus automated (continuous/online) approaches. Panelists stressed that manual sub-hourly sampling is logistically prohibitive, so the development and adoption of reliable online CMD systems was identified as the critical enabler of higher frequency monitoring.

###  Domain 8: core reporting items

The 2014 consensus [2] identified 6 essential data reporting elements; our panel developed more comprehensive guidance, reflecting the increased complexity of CMD research and the need for standardized documentation to enable cross-study comparisons and clinical implementation. Panelists reached consensus on all proposed items:

8.1 | *Catheter type* [100% (37/37)]

8.2 | *Probe membrane length* [94.6% (35/37)]

8.3 | *Perfusion fluid composition* [100% (37/37)]

8.4 | *Perfusion flow rate* [100% (37/37)]

8.5 | *Mechanism used for catheter placement (e.g.*,* twist drill hole*,* transcranial bolt*,* during open craniotomy or craniectomy)* [97.3% (36/37)]

8.6 | *Lesion-relative catheter location*,* confirmed by imaging* [100% (37/37)]

8.7 | *Depth of CMD catheter tip* [82.9% (29/35)]

8.7 Notes | Catheter depth may help infer position in gray versus white matter, although imaging is paramount for precise location.

8.8 | *Time from ictus* [100% (37/37)]

8.8 Notes | While “time from ictus” is standard for SAH/stroke, “time from injury” is preferred for TBI; reporting should use the appropriate terminology for the pathology.

8.9 | *Time from operation (if applicable)* [97.3% (36/37)]

8.10 | *Duration of adequate CMD system (i.e.*,* machine*,* catheter*,* etc.) functioning and total duration of CMD monitoring* [94.3% (33/35)]

8.11 | *Handling of early samples (e.g., if the first hour or two of samples were discarded)* [97.3% (36/37)]

8.12 | *Handling of values outside analyzer limits of detection, missing values, and artifacts* [97.3% (36/37)]

8.13 | *Method of aligning multimodal data streams with different sampling frequencies (e.g., windowed averaging, nearest-neighbor sampling, weighted averaging)* [88.6% (31/35)]

8.14 | *Other concurrent monitoring modalities* [100% (36/36)]

8.15 | *Neuroprotective procedures during monitoring* [89.2% (33/37)]

8.16 | *Complications associated with probe placement or removal* [97.3% (36/37)]

8.17 | *Reference values used for each monitored CMD parameter (e.g.: 0.2 mM, 0.8 mM, or 1.0 mM for brain glucose; 25 or 40 for LPR; etc.)* [94.3% (33/35)]

8.17 Notes | “Reference values” refers to the thresholds used to indicate clinical significance or guide intervention decisions.

8.18 | *If available, plasma glucose records in conjunction with cerebral glucose records* [94.1% (32/34)]

8.19 | *If intravenous glucose supplementation is provided, the rate (mmol/hour) and concentration of the supplementation alongside the set of, if available, associated plasma and cerebral glucose records* [88.6% (31/35)]

### Domain 9: barriers to clinical implementation

The panel agreed on the following obstacles to CMD adoption and successful clinical implementation, acknowledging that some barriers are center-dependent:

9.1 | *Financial constraints* [91.9% (34/37)]

9.2 | *Training of staff on operating procedures* [81.1% (30/37)]

9.3 | *Time demand on staff from handling CMD samples (usually hourly)* [89.2% (33/37)]

9.4 | *Lack of buy-in for CMD catheter placement* [83.3% (30/36)]

9.5 | *Integration of CMD trends with other multimodal device outputs* [78.4% (29/37)]

9.6 | *Difficulty of interpreting high-dimensional data* [75.7% (28/37)]

9.7 | *Lack of automated streaming of CMD data to electronic medical records* [97.1% (34/35)]

9.8 | *Limiting preconception of CMD being exclusive to research applications* [83.8% (31/37)]

9.9 | *Limiting preconception that CMD usage does not affect patient outcomes* [86.5% (32/37)]

9.10 | *Lack of approval of CMD for routine clinical use by some regulatory agencies* [91.4% (32/35)]

While all barriers achieved consensus, one panelist emphasized that the lack of randomized controlled trial evidence demonstrating that CMD improves outcomes is the most fundamental obstacle; robust outcome data would likely diminish the significance of logistical barriers such as cost and training.

## Discussion

This consensus statement provides updated guidance for use of CMD in critical care and serves as a snapshot in time of CMD expert opinion. This guidance is intended to supplement existing clinical management frameworks for acute brain injury by providing CMD-specific consensus that can be integrated alongside established protocols. The robustness of this consensus is supported by a rigorous modified Delphi process characterized by high panel retention and adherence to a priori consensus definitions. The panel largely reaffirmed prior recommendations [2] while adding further nuance in select areas. This consistency of expert opinion over the past decade suggests that foundational elements of CMD practice remain well-grounded.

The extension of guidance to ICH warrants particular consideration. Only 37.8% of panelists (14/37) had clinical experience with ICH (compared to 100% with TBI and 75.7% with SAH), and panelists with clinical ICH experience comprised 33.3–35.3% of non-abstaining respondents across the ICH-specific statements. A methodological choice of this Delphi process was that panelists were not restricted from voting on statements outside their direct clinical experience; rather, panelists were expected to draw upon the relevant literature as well as, where applicable, their CMD experience in related conditions. This was done to maximize response rates, with panelists offered the option to abstain from any statement for which they lacked relevant experience, knowledge, or confidence. This approach applied broadly (e.g., panelists without clinical SAH experience could vote on SAH-related statements, and researchers without clinical experience could vote on clinically oriented statements), but is most consequential for ICH given that this consensus introduces formal ICH guidance for the first time with a minority of panelists having relevant clinical experience. To assess whether this introduced bias, we examined agreement rates among panelists with versus without clinical ICH experience (reported in the Notes for Statements 1.2, 1.3, 3.2, and 5.7). Ultimately, achievement of consensus (≥ 75% aggregate agreement) on ICH-related statements was not driven by extrapolation from panelists without clinical ICH experience. Nonetheless, the ICH-related guidance should be interpreted cautiously given the emerging evidence base.

Two statements did not reach the a priori consensus threshold of ≥ 75% agreement: Statement 4.7 (glutamate–outcome association, 73.5%) and Statement 5.7 (CMD during ICH evacuation, 60.6%). Non-consensus in these areas reflects the current state of the evidence, including uncertainty surrounding the prognostic significance of glutamate elevations and the role of CMD following ICH evacuation, rather than disagreement with the underlying clinical rationale. Indeed, although not reaching consensus, both statements received majority agreement.

The 2014 consensus identified 6 essential data reporting elements. Our panel developed more comprehensive guidance (19 items), aiming to enable multi-center collaboration and facilitate pooled analyses by addressing elements frequently omitted from publications. The suggested reporting items are intended as guidance for best practice rather than mandatory requirements, but adherence will aid external interpretation and reproducibility. Additionally, our identification of 10 implementation barriers addresses a gap in previous guidance, as recognizing obstacles to adoption is essential for progress.

Several considerations warrant acknowledgment. While our panel represented nine countries, the highest representation was from the United Kingdom, United States, and Sweden, which reflects the geographic concentration of active CMD practice and publication; nonetheless, perspectives from other regions, particularly less resource-capable settings, may be under-represented. Furthermore, while high numerical consensus was achieved, qualitative feedback often reflected greater nuance than voting statistics alone suggest; we have attempted to capture this granularity in the accompanying notes. Additionally, unlike the 2014 consensus, which established specific pathological thresholds (e.g., glucose < 0.8 mM; LPR > 25 and > 40), this consensus effort focused on conceptual principles rather than prescriptive numerical cut-offs. Thresholds appearing in our statements are presented as illustrative examples rather than formally updated, consensus-validated values. This approach reflects greater weight placed on temporal trends, multimodal context, lesion-relative catheter location, substantial practice variation across institutions, and flexibility for individualized clinical decision-making. Statement 8.17, which recommends reporting the reference values used for clinical interpretation, accounts for institutional variability. Finally, definitive evidence linking CMD-guided management to improved patient outcomes awaits well-designed, preferably randomized interventional trials. Nonetheless, the panel affirms that the unique metabolic insight provided by CMD supports its continued role in critical care.

## Conclusion

This consensus on the implementation of CMD in critical care provides updated, evidence-informed recommendations across nine domains. Substantial continuity with the 2014 consensus reflects stable expert agreement on foundational CMD practice. Advances include cautious extension of guidance to ICH, expanded reporting standards addressing commonly omitted elements, and identification of clinical implementation barriers. Demonstrating that CMD-guided management improves patient outcomes remains a critical next step for the field.

## Supplementary Information

Below is the link to the electronic supplementary material.

Additional file 1: (.pdf) Supplementary Materials. This file contains Supplement 1 (Survey instruments used across the three Delphi rounds) and Supplement 2 (Complete round-by-round quantitative statement results and consensus calculations).Additional file 1:Additional file 1:

## Data Availability

All data generated or analyzed during this study are included in this published article and its supplementary information files (see Additional file 1).

## Abbreviations

CBF: Cerebral blood flow
CMD: Cerebral microdialysis
CPP: Cerebral perfusion pressure
ICH: Intracerebral hemorrhage
ICP: Intracranial pressure
LPR: Lactate/pyruvate ratio
NBO: Normobaric hyperoxia
PbtO_2_: Brain tissue oxygenation
RBCT: Red blood cell transfusion
SAH: Subarachnoid hemorrhage
TBI: Traumatic brain injury
