# Supplementary material for: Consensus statement from the 2025 Delphi panel on cerebral microdialysis in critical care
Source: Crit Care. 2026 Apr 13;30:262. doi: 10.1186/s13054-026-05993-z (PMC13185327; doi:10.1186/s13054-026-05993-z)
Supplement: Supplementary file 1 — Additional file 1: (.pdf) Supplementary Materials. This file contains Supplement 1 (Survey instruments used across the three Delphi rounds) and Supplement 2 (Complete round-by-round quantitative statement results and consensus calculations).Additional file 1:Additional file 1: [file 13054_2026_5993_MOESM1_ESM.pdf]

Article: Consensus statement from the 2025 Delphi panel on cerebral microdialysis in critical care

Journal: *Critical Care*

Authors: Adel Helmy†, Michael S Baker Jr*,†, Patrick M Chen, Aoife Quinn, Ibrahim Jalloh, Louise Roberts, Neeraj Badjatia, Antonio Belli, Martyn G Boutelle, M Ross Bullock, Jan Claassen, JP Coles, Claire Dahyot-Fizelier, Ari Ercole, Brandon Foreman, Clare Gallagher, Emily J Gilmore, Arun K Gupta, Deepak Gupta, Raimund Helbok, Peter Leroux, Sandra Magnoni, Halinder S Mangat, Niklas Marklund, Anna Teresa Mazzeo, David K Menon, David W Nelson, Virginia Newcombe, Mauro Oddo, Kristine O'Phelan, Patrizio Petrone, Maria A Poca, Ava M Puccio, Claudia S Robertson, Elham Rostami, Juan Sahuquillo, Matthew G Stovell, Anthony J Strong, Teodor Svedung Wettervik, Eric P Thelin, Ivan S Timofeev, Ramon Torné, Alex Valadka, Sara Venturini, Paul Vespa, Chisomo Zimphango, Keri LH Carpenter, Jefferson W Chen, Peter J Hutchinson

†These authors contributed equally and share first authorship.

***Corresponding author**: Michael S Baker Jr (Division of Neurosurgery, Department of Clinical Neurosciences, University of Cambridge, Cambridge, United Kingdom; msb74@cam.ac.uk)

Supplementary materials

Supplement 1: survey instruments **1**

Round 1 survey **1**

Round 2 survey **15**

Round 3 survey **24**

Supplement 2: quantitative statement results **28**

Round 1 results **28**

Round 2 results **34**

Round 3 results **38**

Supplement 1: survey instruments

Round 1 survey

Panelist registration and demographics

Before proceeding to the consensus statements, panelists completed the following registration and demographic questions:

1. Full name
2. Consent to citation as a member of the Delphi panel in the final consensus statement paper (Yes/No)
3. Email address
4. Gender (Male/Female/Other)
5. Primary country of cerebral microdialysis (CMD) experience
6. Role(s) in which you have CMD experience (Nurse/Researcher/Physician/Other, with specialty specification)
7. Years of CMD experience
8. Clinical experience (Yes/No, with years if applicable)
    - If yes: Condition(s) for which you have clinical experience (TBI/SAH/ICH)
    - If yes: Probe placement experience (TBI/SAH/ICH/Other condition)

Consensus statements

Response scale: Strongly Agree | Agree | Neutral | Disagree | Strongly Disagree | Not Applicable / Decline to Respond

For each statement, panelists were invited to provide qualitative feedback on how the statement could be modified to increase agreement. Statements that did not achieve consensus would be revised based on this feedback for Round 2. Statement consensus was defined a priori as ≥75% agreement (Strongly Agree + Agree) among non-abstaining respondents, with a minimum of 30 non-abstaining respondents required per statement.

Additionally, panelists were invited to suggest entirely new statements to be considered for voting in Round 2. This was the only round in which new statement suggestions were solicited.

Statement 1

Statement: *Cerebral microdialysis (CMD) monitoring should be considered for the following condition(s) when there is a concurrent indication for invasive cerebral monitoring (e.g., intracranial pressure) or when access is available at the time of neurosurgical procedure: (1) traumatic brain injury (TBI); (2) subarachnoid hemorrhage (SAH); (3) intracerebral hemorrhage (ICH).*

Context: We are seeking a formal inclusion formula for identifying patients for whom CMD is most suitable / beneficial. Establishing selection criteria is meant to maximize the benefit of monitoring while mitigating unnecessary complications from monitoring in inappropriately selected patients.

Statement 2

Statement: *Consultant or attending neurosurgeons or appropriately trained intensivists should be responsible for CMD catheter placement in order to avoid adverse events and deal with any complications.*

Context: This statement proposes professional qualifications for probe placement so as to optimize safety and success.

Statement 3

Statement: *Dependent on local availability and expertise, CMD catheters can be placed by twist drill hole, transcranial bolt, or during open craniotomy or craniectomy, with each of these procedures being safe and effective.*

Context: Recent studies have shown the safety of CMD catheter insertion via single burr hole,[1] quad-lumen bolt and craniotomy,[2] and triple-lumen bolt.[3]

References:

1. Foreman B, Ngwenya LB, Stoddard E, Hinzman JM, Andaluz N, Hartings JA. Safety and Reliability of Bedside, Single Burr Hole Technique for Intracranial Multimodality Monitoring in Severe Traumatic Brain Injury. Neurocrit Care. 2018;29(3):469-480. doi:10.1007/s12028-018-0551-7
2. Falcone JA, Chen JW. Technical notes on the placement of cerebral microdialysis: A single center experience. Front Neurol. 2023;13:1041952. Published 2023 Jan 9. doi:10.3389/fneur.2022.1041952
3. Bailey RL, Quattrone F, Curtin C, et al. The Safety of Multimodality Monitoring Using a Triple-Lumen Bolt in Severe Acute Brain Injury. World Neurosurg. 2019;130:e62-e67. doi:10.1016/j.wneu.2019.05.195

Statement 4

Statement: *The first hour of microdialysate should not be used to make clinical decisions due to the confounders of catheter insertion and pump flushing.*

Context: There is variation in whether the first hour or the first two hours of microdialysate are disregarded in analyses and clinical decision making. Panelist responses may afford insight into the optimal amount of time to disregard.

Statement 5

Statement: *Focally recorded CMD data may be generalizable to the whole brain depending on the location of the catheter with respect to any focal lesions and the pattern of brain injury.*

Context: It is well-established that CMD should be primarily used for insight on focal brain chemistry near the probe. However, much evidence has been produced for the differences in baseline CMD values from perilesional to normal appearing brain (in cases of TBI,[1,2] SAH,[3-6] and ICH).[7] A potential application of this knowledge is to be able to infer something about other regions of the brain given these predictable differences (e.g., if catheter is in perilesional tissue, then you would expect lower LPR in non-injured tissue) and make assumptions about the whole brain or other parts of it. The importance of also taking into account the diagnosis, even as slight of a difference as traumatic vs. spontaneous ICH, was recently made evident.[8]

References:

1. Timofeev I, Czosnyka M, Carpenter KL, et al. Interaction between brain chemistry and physiology after traumatic brain injury: impact of autoregulation and microdialysis catheter location. J Neurotrauma. 2011;28(6):849-860. doi:10.1089/neu.2010.1656

2. Engström M, Polito A, Reinstrup P, et al. Intracerebral microdialysis in severe brain trauma: the importance of catheter location. J Neurosurg. 2005;102(3):460-469. doi:10.3171/jns.2005.102.3.0460
3. Kofler M, Gaasch M, Rass V, et al. The Importance of Probe Location for the Interpretation of Cerebral Microdialysis Data in Subarachnoid Hemorrhage Patients. Neurocrit Care. 2020;32(1):135-144. doi:10.1007/s12028-019-00713-8
4. Svedung Wettervik T, Engquist H, Hånell A, et al. Cerebral Microdialysis Monitoring of Energy Metabolism: Relation to Cerebral Blood Flow and Oxygen Delivery in Aneurysmal Subarachnoid Hemorrhage. J Neurosurg Anesthesiol. 2023;35(4):384-393. doi:10.1097/ANA.0000000000000854
5. Torné R, Culebras D, Sanchez-Etayo G, et al. Double hemispheric Microdialysis study in poor-grade SAH patients. Sci Rep. 2020;10(1):7466. Published 2020 May 4. doi:10.1038/s41598-020-64543-x
6. Tholance Y, Barcelos GK, Perret-Liaudet A, et al. Placing intracerebral probes to optimise detection of delayed cerebral ischemia and allow for the prediction of patient outcome in aneurysmal subarachnoid haemorrhage. J Cereb Blood Flow Metab. 2017;37(8):2820-2832. doi:10.1177/0271678X16675880
7. Tobieson L, Ghafouri B, Zsigmond P, Rossitti S, Hillman J, Marklund N. Dynamic protein changes in the perihaemorrhagic zone of Surgically Treated Intracerebral Haemorrhage Patients. Sci Rep. 2019;9(1):3181. Published 2019 Feb 28. doi:10.1038/s41598-019-39499-2
8. Kang J, Shah I, Shahrestani S, et al. Friedman's Gradient-Boosting Algorithm Predicts Lactate-Pyruvate Ratio Trends in Cases of Intracerebral Hemorrhages. World Neurosurg. 2024;187:e620-e628. doi:10.1016/j.wneu.2024.04.136

Statement 6

Statement: *Use of multiple microdialysis catheters is safe and adds value with more robust insight than single catheter monitoring in cases where several patterns of injury occur in the same patient.*

Context: Multiple catheters in differing locations of a patient's brain may help paint a better picture of the whole brain's condition. This was suggested as feasible in our previous consensus statement,[1] and it is worth assessing current enthusiasm of its merits. Falcone and Chen[2] describe the benefit of having a 'control' probe in normal appearing brain tissue and another probe in perilesional tissue in cases of focal TBI. Furthermore, use of multiple CMD monitors has been shown to be safe and beneficial in SAH patients.[3]

References:

1. Hutchinson PJ, Jalloh I, Helmy A, et al. Consensus statement from the 2014 International Microdialysis Forum. Intensive Care Med. 2015;41(9):1517-1528. doi:10.1007/s00134-015-3930-y
2. Falcone JA, Chen JW. Technical notes on the placement of cerebral microdialysis: A single center experience. Front Neurol. 2023;13:1041952. Published 2023 Jan 9. doi:10.3389/fneur.2022.1041952

3. Torné R, Culebras D, Sanchez-Etayo G, et al. Double hemispheric Microdialysis study in poor-grade SAH patients. Sci Rep. 2020;10(1):7466. Published 2020 May 4. doi:10.1038/s41598-020-64543-x

Statement 7

Statement: *Integration of CMD data with multimodal physiological signals is essential for holistic clinical decision making.*

Context: We believe that CMD should be used in tandem with other intracranial modalities such as ICP and PbtO2 for optimal understanding of acute injured brain physiology, and that integration of signals into a consolidated interface is key to interpretation and taking action.

Statement 8

Statement: *CMD can provide early indication of impending deterioration and secondary injury such as: (1) intracranial hypertension in TBI; (2) symptomatic delayed ischemia in SAH; (3) cerebral edema in the perihematomal zone of ICH (intracerebral hemorrhage).*

Context: Our previous consensus statement[1] cites multiple papers showing CMD's power to predict secondary insults including intracranial hypertension for TBI and symptomatic delayed ischemia for SAH.[2-5] The use of CMD for ICH (intracerebral hemorrhage) is more nascent, but a potential application is for early detection of secondary insults such as edema.

References:

1. Hutchinson PJ, Jalloh I, Helmy A, et al. Consensus statement from the 2014 International Microdialysis Forum. Intensive Care Med. 2015;41(9):1517-1528. doi:10.1007/s00134-015-3930-y
2. Adamides AA, Rosenfeldt FL, Winter CD, et al. Brain tissue lactate elevations predict episodes of intracranial hypertension in patients with traumatic brain injury. J Am Coll Surg. 2009;209(4):531-539. doi:10.1016/j.jamcollsurg.2009.05.028
3. Belli A, Sen J, Petzold A, et al. Metabolic failure precedes intracranial pressure rises in traumatic brain injury: a microdialysis study. Acta Neurochir (Wien). 2008;150(5):461-470. doi:10.1007/s00701-008-1580-3
4. Skjøth-Rasmussen J, Schulz M, Kristensen SR, Bjerre P. Delayed neurological deficits detected by an ischemic pattern in the extracellular cerebral metabolites in patients with aneurysmal subarachnoid hemorrhage. J Neurosurg. 2004;100(1):8-15. doi:10.3171/jns.2004.100.1.0008
5. Sarrafzadeh AS, Sakowitz OW, Kiening KL, et al. Bedside microdialysis: a tool to monitor cerebral metabolism in subarachnoid hemorrhage patients? Crit Care Med. 2002;30(5):1062-1070. doi:10.1097/00003246-200205000-00018

Statement 9

Statement: *In a hierarchical approach to treating deranged metabolism using a CMD-inclusive multimodal monitoring scheme: (1) ICP should be addressed with priority (target $\leq$ 20 mmHg); (2) then PbtO2 (target $\geq$ 15 mmHg); (3) and finally, brain glucose (target $\geq$ 1.0 mM).*

Context: We have laid groundwork for a tiered approach to addressing deranged metabolism.[1,2] A target of PbtO2 ≥ 15 mmHg is supported by a study showing reduced evidence of tissue hypoxia (measured by [18F]FMISO trapping) in this range.[3] Additionally, there has long been evidence of the association of PbtO2 ≤ 15 mmHg with mortality.[4]

References:

1. Thelin EP, Carpenter KL, Hutchinson PJ, Helmy A. Microdialysis Monitoring in Clinical Traumatic Brain Injury and Its Role in Neuroprotective Drug Development. AAPS J. 2017;19(2):367-376. doi:10.1208/s12248-016-0027-7
2. Khellaf A, Garcia NM, Tajsic T, et al. Focally administered succinate improves cerebral metabolism in traumatic brain injury patients with mitochondrial dysfunction. J Cereb Blood Flow Metab. 2022;42(1):39-55. doi:10.1177/0271678X211042112
3. Veenith TV, Carter EL, Geeraerts T, et al. Pathophysiologic Mechanisms of Cerebral Ischemia and Diffusion Hypoxia in Traumatic Brain Injury. JAMA Neurol. 2016;73(5):542-550. doi:10.1001/jamaneurol.2016.0091
4. Valadka AB, Gopinath SP, Contant CF, Uzura M, Robertson CS. Relationship of brain tissue PO2 to outcome after severe head injury. Crit Care Med. 1998;26(9):1576-1581. doi:10.1097/00003246-199809000-00029

Statement 10

Statement: *For those patients with abnormally high LPR (> 25) detected by CMD despite other multimodal parameters having been normalized in line with locally determined treatment protocols: (1) Ischemia is indicated by concurrently insufficient substrate (e.g., brain glucose < 1.0 mM or pyruvate < 70 µM); (2) Clinical mitochondrial dysfunction is indicated by concurrently sufficient substrate (e.g., brain glucose ≥ 1.0 mM and pyruvate ≥ 70 µM).*

Context: In a recent systematic review,[1] we found that the field describes raised LPR using an umbrella term of 'metabolic crisis' which can be split into ischemia, when substrates such as glucose, pyruvate, or oxygen are insufficient, and mitochondrial dysfunction, when substrates for aerobic metabolism are sufficient but inadequately utilized. However, we believe that there is a need to normalize other multimodal parameters in order to identify these states accurately. Because an LPR above 25 correlates with a worse clinical outcome while a higher threshold of 40 does not show a more significant relationship,[2] we suggest use of the threshold of 25 for metabolic crises. For sufficient substrate levels, a glucose threshold of 1.0 mM is chosen for consistency with our definition of neuroglycopenia as brain glucose < 1.0 mM,[3,4] and a pyruvate threshold of 70 µM is chosen because this encapsulates the thresholds suggested by other groups for sufficient substrate delivery (70 µM[5,6] and 120 µM).[7,8]

References:

1. Venturini S, Bhatti F, Timofeev I, et al. Microdialysis-Based Classifications of Abnormal Metabolic States after Traumatic Brain Injury: A Systematic Review of the Literature. J Neurotrauma. 2023;40(3-4):195-209. doi:10.1089/neu.2021.0502
2. Timofeev I, Carpenter KL, Nortje J, et al. Cerebral extracellular chemistry and outcome following traumatic brain injury: a microdialysis study of 223 patients. Brain. 2011;134(Pt 2):484-494. doi:10.1093/brain/awq353

3. Thelin EP, Carpenter KL, Hutchinson PJ, Helmy A. Microdialysis Monitoring in Clinical Traumatic Brain Injury and Its Role in Neuroprotective Drug Development. AAPS J. 2017;19(2):367-376. doi:10.1208/s12248-016-0027-7
4. Khellaf A, Garcia NM, Tajsic T, et al. Focally administered succinate improves cerebral metabolism in traumatic brain injury patients with mitochondrial dysfunction. J Cereb Blood Flow Metab. 2022;42(1):39-55. doi:10.1177/0271678X211042112
5. Gupta D, Singla R, Mazzeo AT, et al. Detection of metabolic pattern following decompressive craniectomy in severe traumatic brain injury: A microdialysis study. Brain Inj. 2017;31(12):1660-1666. doi:10.1080/02699052.2017.1370553
6. Nordström CH, Nielsen TH, Schalén W, Reinstrup P, Ungerstedt U. Biochemical indications of cerebral ischaemia and mitochondrial dysfunction in severe brain trauma analysed with regard to type of lesion. Acta Neurochir (Wien). 2016;158(7):1231-1240. doi:10.1007/s00701-016-2835-z
7. Svedung Wettervik T, Engquist H, Howells T, et al. Arterial Oxygenation in Traumatic Brain Injury-Relation to Cerebral Energy Metabolism, Autoregulation, and Clinical Outcome. J Intensive Care Med. 2021;36(9):1075-1083. doi:10.1177/0885066620944097
8. Marini CP, Stoller C, McNelis J, Del Deo V, Prabhakaran K, Petrone P. Correlation of brain flow variables and metabolic crisis: a prospective study in patients with severe traumatic brain injury. Eur J Trauma Emerg Surg. 2022;48(1):537-544. doi:10.1007/s00068-020-01447-5

Statement 11

Statement: *The most reliable clinical CMD parameters are glucose and LPR, which provide more clinically actionable information than monitoring glutamate or glycerol.*

Context: The utility of glucose and LPR over glutamate and glycerol was established in our previous consensus statement,[1] and this is now being reassessed to see if expert opinion has changed on the topic.

References:

1. Hutchinson PJ, Jalloh I, Helmy A, et al. Consensus statement from the 2014 International Microdialysis Forum. Intensive Care Med. 2015;41(9):1517-1528. doi:10.1007/s00134-015-3930-y

Statement 12

Statement: *Absolute lactate and pyruvate concentrations, alongside glucose, should be considered when interpreting heightened LPR, as this can indicate if the abnormality relates to a failure of metabolic substrate delivery.*

Context: We seek consensus surrounding the topic of considering analyte concentrations when interpreting elevated LPR. The Cambridge group has suggested cerebral glucose to be taken into account when interpreting elevated LPR, specifically when identifying mitochondrial dysfunction.[1,2] Contrastingly, other groups suggest lactate[3] or pyruvate[4-7] to be taken into account when identifying ischemia vs. mitochondrial dysfunction.

References:

1. Thelin EP, Carpenter KL, Hutchinson PJ, Helmy A. Microdialysis Monitoring in Clinical Traumatic Brain Injury and Its Role in Neuroprotective Drug Development. AAPS J. 2017;19(2):367-376. doi:10.1208/s12248-016-0027-7
2. Khellaf A, Garcia NM, Tajsic T, et al. Focally administered succinate improves cerebral metabolism in traumatic brain injury patients with mitochondrial dysfunction. J Cereb Blood Flow Metab. 2022;42(1):39-55. doi:10.1177/0271678X211042112
3. Sahuquillo J, Merino MA, Sánchez-Guerrero A, et al. Lactate and the lactate-to-pyruvate molar ratio cannot be used as independent biomarkers for monitoring brain energetic metabolism: a microdialysis study in patients with traumatic brain injuries [published correction appears in PLoS One. 2014;9(10):e111821]. PLoS One. 2014;9(7):e102540. Published 2014 Jul 15. doi:10.1371/journal.pone.0102540
4. Nordström CH, Nielsen TH, Schalén W, Reinstrup P, Ungerstedt U. Biochemical indications of cerebral ischaemia and mitochondrial dysfunction in severe brain trauma analysed with regard to type of lesion. Acta Neurochir (Wien). 2016;158(7):1231-1240. doi:10.1007/s00701-016-2835-z
5. Gupta D, Singla R, Mazzeo AT, et al. Detection of metabolic pattern following decompressive craniectomy in severe traumatic brain injury: A microdialysis study. Brain Inj. 2017;31(12):1660-1666. doi:10.1080/02699052.2017.1370553
6. Svedung Wettervik T, Engquist H, Howells T, et al. Arterial Oxygenation in Traumatic Brain Injury-Relation to Cerebral Energy Metabolism, Autoregulation, and Clinical Outcome. J Intensive Care Med. 2021;36(9):1075-1083. doi:10.1177/0885066620944097
7. Marini CP, Stoller C, McNelis J, Del Deo V, Prabhakaran K, Petrone P. Correlation of brain flow variables and metabolic crisis: a prospective study in patients with severe traumatic brain injury. Eur J Trauma Emerg Surg. 2022;48(1):537-544. doi:10.1007/s00068-020-01447-5

Statement 13

Statement: *Brain glucose as recorded from non-injured tissue can be manipulated in a controlled, predictable manner in the traumatically injured brain (e.g., by insulin infusion or glucose administration), as it holds a positive linear relationship with plasma glucose.*

Context: Our previous consensus statement[1] cited the positive linear relationship between peripheral glucose and non-injured brain tissue glucose levels in TBI patients.[2] We want to see if there has been any change in the field's conception of this relationship.

References:

1. Hutchinson PJ, Jalloh I, Helmy A, et al. Consensus statement from the 2014 International Microdialysis Forum. Intensive Care Med. 2015;41(9):1517-1528. doi:10.1007/s00134-015-3930-y
2. Rostami E, Bellander BM. Monitoring of glucose in brain, adipose tissue, and peripheral blood in patients with traumatic brain injury: a microdialysis study. J Diabetes Sci Technol. 2011;5(3):596-604. Published 2011 May 1. doi:10.1177/193229681100500314

Statement 14

Statement: *Administration of 50% dextrose may be used to raise plasma glucose up to 10 mM (180 mg/dL) in an effort to resolve neuroglycopenia (brain glucose < 1.0 mM).*

Context: Given the relationship between plasma glucose and cerebral glucose in non-injured tissue,[1] we would like to solidify guidance for brain glucose manipulation. Since our previous consensus statement,[2] much research has been published on ideal intensity of glycemic control and impact on mortality and neurological outcome.[3] Although results are inconsistent,[4] conventional glycemic control does seem less associated with neuroglycopenia.[5-7] For context, conventional glycemic control allows for a higher maximum plasma glucose of 10 mM than tight glycemic control (between 4-7 mM). We use a threshold for neuroglycopenia of 1.0 mM,[8,9] although < 0.2 mM has been suggested as a critical threshold.[2]

References:

1. Rostami E, Bellander BM. Monitoring of glucose in brain, adipose tissue, and peripheral blood in patients with traumatic brain injury: a microdialysis study. J Diabetes Sci Technol. 2011;5(3):596-604. Published 2011 May 1. doi:10.1177/193229681100500314
2. Hutchinson PJ, Jalloh I, Helmy A, et al. Consensus statement from the 2014 International Microdialysis Forum. Intensive Care Med. 2015;41(9):1517-1528. doi:10.1007/s00134-015-3930-y
3. Garcia-Ballestas E, Villafañe J, Nuñez-Baez K, et al. A systematic review and meta-analysis on glycemic control in traumatic brain injury. Clin Neurol Neurosurg. 2024;245:108504. doi:10.1016/j.clineuro.2024.108504
4. Gribnau A, van Zuylen ML, Coles JP, Plummer MP, Hermanns H, Hermanides J. Cerebral Glucose Metabolism following TBI: Changes in Plasma Glucose, Glucose Transport and Alternative Pathways of Glycolysis-A Translational Narrative Review. Int J Mol Sci. 2024;25(5):2513. Published 2024 Feb 21. doi:10.3390/ijms25052513
5. Oddo M, Schmidt JM, Carrera E, et al. Impact of tight glycemic control on cerebral glucose metabolism after severe brain injury: a microdialysis study. Crit Care Med. 2008;36(12):3233-3238. doi:10.1097/CCM.0b013e31818f4026
6. NICE-SUGAR Study Investigators, Finfer S, Chittock DR, et al. Intensive versus conventional glucose control in critically ill patients. N Engl J Med. 2009;360(13):1283-1297. doi:10.1056/NEJMoa0810625
7. Plummer MP, Notkina N, Timofeev I, Hutchinson PJ, Finnis ME, Gupta AK. Cerebral metabolic effects of strict versus conventional glycaemic targets following severe traumatic brain injury. Crit Care. 2018;22(1):16. Published 2018 Jan 25. doi:10.1186/s13054-017-1933-5
8. Thelin EP, Carpenter KL, Hutchinson PJ, Helmy A. Microdialysis Monitoring in Clinical Traumatic Brain Injury and Its Role in Neuroprotective Drug Development. AAPS J. 2017;19(2):367-376. doi:10.1208/s12248-016-0027-7
9. Khellaf A, Garcia NM, Tajsic T, et al. Focally administered succinate improves cerebral metabolism in traumatic brain injury patients with mitochondrial dysfunction. J Cereb Blood Flow Metab. 2022;42(1):39-55. doi:10.1177/0271678X211042112

Statement 15

Statement: *Despite the association of increased cerebral glutamate with brain glucose > 5.0 mM, there is insufficient evidence to define a critical upper limit for brain glucose.*

Context: Although discussion of an optimal range of brain glucose pervades the literature,[1] thus far there is insufficient evidence to define a critical upper limit for brain glucose. Meierhans et al[2] have shown that glutamate is higher with brain glucose > 5.0 mM. However, they also concluded that this is not enough to warrant an avoidance of brain glucose concentrations above 5.0 mM. Furthermore, the association between LPR and glucose was only shown to be well-defined for brain glucose < 1.0 mM, so cerebral metabolism (LPR) cannot be said to definitively worsen with higher brain glucose values.[3]

References:

1. Kurtz P, Rocha EEM. Nutrition Therapy, Glucose Control, and Brain Metabolism in Traumatic Brain Injury: A Multimodal Monitoring Approach. Front Neurosci. 2020;14:190. Published 2020 Mar 24. doi:10.3389/fnins.2020.00190
2. Meierhans R, Béchir M, Ludwig S, et al. Brain metabolism is significantly impaired at blood glucose below 6 mM and brain glucose below 1 mM in patients with severe traumatic brain injury. Crit Care. 2010;14(1):R13. doi:10.1186/cc8869
3. Guilfoyle MR, Helmy A, Donnelly J, et al. Characterising the dynamics of cerebral metabolic dysfunction following traumatic brain injury: a microdialysis study in 619 patients. PLoS One. 2021;16(12):e0260291. Published 2021 Dec 16. doi:10.1371/journal.pone.0260291

Statement 16

Statement: *Although a CPP of 60-70 mmHg is recommended within Brain Trauma Foundation Guidelines, metabolic state as indicated by CMD has the potential to inform individualization of CPP targets.*

Context: The Brain Trauma Foundation[1] suggests a CPP of 60-70 mmHg and avoiding aggressive attempts to maintain CPP above 70 mmHg. Although CMD is not currently used to personalize CPP targets (refinement with PRx is, however, being explored by certain groups),[2] we want to gauge enthusiasm for doing so in this Delphi study.

References:

1. Carney N, Totten AM, O'Reilly C, et al. Guidelines for the Management of Severe Traumatic Brain Injury, Fourth Edition. Neurosurgery. 2017;80(1):6-15. doi:10.1227/NEU.0000000000001432
2. Tas J, Beqiri E, van Kaam RC, et al. Targeting Autoregulation-Guided Cerebral Perfusion Pressure after Traumatic Brain Injury (COGiTATE): A Feasibility Randomized Controlled Clinical Trial. J Neurotrauma. 2021;38(20):2790-2800. doi:10.1089/neu.2021.0197

Statement 17

Statement: *For optimal safety during episodes of vulnerability indicated by CMD, neurological wake-up tests may not be advisable in patients with a trend of insufficient cerebral glucose (< 1.0 mM) or high LPR (> 25).*

Context: Helbok et al[1] showed wake-up test failure in patients with deranged metabolism. Although the association the group found was only of general trends of lower brain glucose and higher LPR being associated with wake-up test failure, we propose thresholds (25 and 1.0 mM, respectively) for enhanced actionability.

References:

1. Helbok R, Kurtz P, Schmidt MJ, et al. Effects of the neurological wake-up test on clinical examination, intracranial pressure, brain metabolism and brain tissue oxygenation in severely brain-injured patients. Crit Care. 2012;16(6):R226. Published 2012 Nov 27. doi:10.1186/cc11880

Statement 18

Statement: *Disturbance of the brain's metabolic state as indicated by CMD can guide de-escalation of therapy because it demonstrates a remaining vulnerability to physiological perturbations.*

Context: In addition to CMD informing the appropriateness of wake-up tests (and consequently de-escalation),[1] we also believe that CMD may be able to guide de-escalation itself.

References:

1. Helbok R, Kurtz P, Schmidt MJ, et al. Effects of the neurological wake-up test on clinical examination, intracranial pressure, brain metabolism and brain tissue oxygenation in severely brain-injured patients. Crit Care. 2012;16(6):R226. Published 2012 Nov 27. doi:10.1186/cc11880

Statement 19

Statement: *In cases of concurrent Hgb < 9.0 g/dL with PbtO2 < 20 mmHg, the triggering of red blood cell transfusion should be made more likely if LPR > 25 considering the inverse relationship of anemic Hgb and LPR.*

Context: Oddo et al[1] demonstrated the benefit of red blood cell transfusion (RBCT) on cerebral metabolism when Hgb < 9.0 g/dL in cases of aneurysmal SAH. Although packed RBCT was not associated with benefits to metabolism,[2] Kurtz et al[3] found that anemia (Hgb < 9.0 g/dL and Hgb between 9.1-10 g/dL) was associated with risk of LPR > 40. Further supporting a threshold of 9.0 g/dL Hgb, a liberal strategy (Hgb < 9.0 g/dL) for RBCT in acute brain injury was shown to be less associated with ischemia and unfavorable outcomes compared to a restrictive strategy (Hgb < 7.0 g/dL) in a recent randomized controlled trial.[4] The BOOST-3 protocol[5] suggests RBCT as a tier 2 measure for correction of PbtO2 < 20 mmHg.

References:

1. Oddo M, Milby A, Chen I, et al. Hemoglobin concentration and cerebral metabolism in patients with aneurysmal subarachnoid hemorrhage. Stroke. 2009;40(4):1275-1281. doi:10.1161/STROKEAHA.108.527911
2. Kurtz P, Helbok R, Claassen J, et al. The Effect of Packed Red Blood Cell Transfusion on Cerebral Oxygenation and Metabolism After Subarachnoid Hemorrhage. Neurocrit Care. 2016;24(1):118-121. doi:10.1007/s12028-015-0180-3
3. Kurtz P, Schmidt JM, Claassen J, et al. Anemia is associated with metabolic distress and brain tissue hypoxia after subarachnoid hemorrhage. Neurocrit Care. 2010;13(1):10-16. doi:10.1007/s12028-010-9357-y
4. Taccone FS, Rynkowski CB, Møller K, et al. Restrictive vs Liberal Transfusion Strategy in Patients With Acute Brain Injury: The TRAIN Randomized Clinical Trial [published correction appears in JAMA. 2025 Mar 11;333(10):911. doi: 10.1001/jama.2025.1719.]. JAMA. 2024;332(19):1623-1633. doi:10.1001/jama.2024.20424
5. Bernard F, Barsan W, Diaz-Arrastia R, Merck LH, Yeatts S, Shutter LA. Brain Oxygen Optimization in Severe Traumatic Brain Injury (BOOST-3): a multicentre, randomised, blinded-endpoint, comparative effectiveness study of brain tissue oxygen and intracranial pressure monitoring versus intracranial pressure alone. BMJ Open. 2022;12(3):e060188. Published 2022 Mar 10. doi:10.1136/bmjopen-2021-060188

Statement 20

Statement: *CMD may be used adjunctly to assess recovery following ICH resection and indicate the need for further interventions (e.g., EVD placement) in cases of non-responding or worsening condition despite resection.*

Context: ICH is more common than SAH, but CMD is not used as much for ICH. However, novel surgical treatments for ICH and evidence for CMD monitoring's utility in ICH has accumulated since our last consensus statement.[1] The association with outcome of the LPR ipsilateral to spontaneous ICH was shown over a decade ago.[2] Considering the association in spontaneous ICH patients of perihematomal LPR with cerebral autoregulation and outcome, CMD could be used adjunctly to guide care.[3] In fact, recently a trend was established of decreasing LPR following ICH resection.[4] It follows that deviation from this trend should suggest the need for further intervention, and in fact a case study has shown that CMD can indicate refractory pathology despite adequate ICH resection - addressing the refractorily deranged multimodal parameters with placement of an EVD lead to correction of ICP, PbtO2, and metabolic parameters (glucose, pyruvate and the LPR).[5] Thus, CMD may be used adjunctly to assess recovery following hematoma resection and indicate the need for further interventions in cases of non-responding or worsening condition despite resection. Importantly, spontaneous and traumatic ICH each demonstrate distinct perihematomal metabolic trends, signifying a need for stratification in the interpretation of data based on particular ICH diagnosis.[4]

References:

1. Hutchinson PJ, Jalloh I, Helmy A, et al. Consensus statement from the 2014 International Microdialysis Forum. Intensive Care Med. 2015;41(9):1517-1528. doi:10.1007/s00134-015-3930-y

2. Nikaina I, Paterakis K, Paraforos G, et al. Cerebral perfusion pressure, microdialysis biochemistry, and clinical outcome in patients with spontaneous intracerebral hematomas. J Crit Care. 2012;27(1):83-88. doi:10.1016/j.jcrc.2011.04.004
3. Rasulo F, Piva S, Park S, et al. The Association Between Peri-Hemorrhagic Metabolites and Cerebral Hemodynamics in Comatose Patients With Spontaneous Intracerebral Hemorrhage: An International Multicenter Pilot Study Analysis. Front Neurol. 2020;11:568536. Published 2020 Oct 26. doi:10.3389/fneur.2020.568536
4. Kang J, Shah I, Shahrestani S, et al. Friedman's Gradient-Boosting Algorithm Predicts Lactate-Pyruvate Ratio Trends in Cases of Intracerebral Hemorrhages. World Neurosurg. 2024;187:e620-e628. doi:10.1016/j.wneu.2024.04.136
5. Shah I, Chen PM, Tran DKT, Chen JW. Cerebral microdialysis demonstrates improvements in brain metabolism with cerebrospinal fluid diversion in spontaneous intracerebral hemorrhage. Surg Neurol Int. 2023;14:395. Published 2023 Nov 10. doi:10.25259/SNI_679_2023

Statement 21

Statement: *TBI presenting with concomitant lactate > 3.5 mM and pyruvate < 120 µM indicates the utility of short (e.g., 2 hours) normobaric hyperoxia (100% FiO2, achieving pO2 > 12 kPa) as a therapeutic trial to mitigate deranged cerebral metabolism.*

Context: We believe that normobaric hyperoxia (NBO) may only be recommend for TBI patients. Most NBO-CMD studies have been for TBI patients, so there is insufficient evidence to warrant use for SAH or ICH given the risks.[1] Furthermore, it seems to be most beneficial given concomitant pyruvate < 120 µM[2] and lactate > 3.5 mM.[3,4] The most common method is raising FiO2 directly to 100%.[1] It seems that 2 hours is reasonable for an NBO trial, considering the residual benefit to cerebral metabolism post-challenge, although it is not clear how long this benefit persists after NBO.[3,5] Furthermore, longer challenges may not be warranted considering the potential for concurrent oxidative stress exacerbation.[4]

References:

1. Giannì G, Minini A, Fratino S, et al. The Impact of Short-Term Hyperoxia on Cerebral Metabolism: A Systematic Review and Meta-Analysis. Neurocrit Care. 2022;37(2):547-557. doi:10.1007/s12028-022-01529-9
2. Svedung Wettervik T, Engquist H, Howells T, et al. Arterial Oxygenation in Traumatic Brain Injury-Relation to Cerebral Energy Metabolism, Autoregulation, and Clinical Outcome. J Intensive Care Med. 2021;36(9):1075-1083. doi:10.1177/0885066620944097
3. Vilalta A, Sahuquillo J, Merino MA, et al. Normobaric hyperoxia in traumatic brain injury: does brain metabolic state influence the response to hyperoxic challenge?. J Neurotrauma. 2011;28(7):1139-1148. doi:10.1089/neu.2010.1720
4. Vidal-Jorge M, Sánchez-Guerrero A, Mur-Bonet G, et al. Does Normobaric Hyperoxia Cause Oxidative Stress in the Injured Brain? A Microdialysis Study Using 8-Iso-Prostaglandin F2α as a Biomarker. J Neurotrauma. 2017;34(19):2731-2742. doi:10.1089/neu.2017.4992

5. Ghosh A, Highton D, Kolyva C, Tachtsidis I, Elwell CE, Smith M. Hyperoxia results in increased aerobic metabolism following acute brain injury. J Cereb Blood Flow Metab. 2017;37(8):2910-2920. doi:10.1177/0271678X16679171

Statement 22

Statement: *Hourly CMD sampling is sufficient to add clinical benefit.*

Context: This statement addresses the value of hourly sampling.

Statement 23

Statement: *Higher frequency sampling (sub-hourly) or continuous monitoring may increase the utility of CMD as online monitoring systems are developed.*

Context: This statement addresses the potential role of higher frequency sampling.

Statement 24

Statement: *Core reporting items include: (1) mechanism used for catheter placement (e.g., twist drill hole, transcranial bolt, during open craniotomy or craniectomy); (2) catheter type; (3) probe membrane length; (4) lesion-relative catheter location confirmed by imaging; (5) perfusion fluid composition; (6) perfusion flow rate; (7) handling of early samples (e.g., if the first hour or two of samples were discarded); (8) handling of values outside of analyzer limits of detection, missing values, and artifacts; (9) other concurrent monitoring modalities; (10) method of aligning multimodal data streams with different sampling frequencies (e.g., windowed averaging, nearest-neighbor sampling, weighted averaging); (11) neuroprotective procedures during monitoring; (12) time from ictus; (13) time from operation (if applicable); (14) complications associated with probe placement or removal.*

Context: This section seeks to establish a set of items that should be recorded by standard in order to ensure meaningfulness of data and aid in communication of CMD results.

Statement 25

Statement: *Currently, the largest barriers to clinical implementation of CMD monitoring are center-dependent, but generally include: (1) financial constraints; (2) training of staff on operating procedures; (3) lack of buy-in for CMD catheter placement; (4) the time demand on staff from the handling of CMD samples (usually hourly); (5) integration of CMD trends with other multimodal device outputs; (6) lack of ability to stream CMD data to electronic medical records; (7) difficulty of interpreting high dimensional data; (8) a limiting preconception of CMD being exclusive to research applications; (9) a limiting preconception that CMD usage does not affect outcomes.*

Context: We would like to place a large emphasis on barriers to implementation for this consensus statement so that appropriate solutions to those barriers may be found. This survey item is key in understanding the obstacles that centers face in implementing CMD.

Round 2 survey

Round 2 presented revised statements based on Round 1 feedback. Statements that did not achieve consensus in Round 1 were revised according to panelist suggestions. New statements suggested by panelists during Round 1 were also included.

Opening questions

1. Full name — as it should appear on any publication
2. Preferred email address

Consensus statements

Response scale: Strongly Agree | Agree | Neutral | Disagree | Strongly Disagree | Decline to Respond

For each statement, panelists were again invited to provide qualitative feedback on how the statement could be modified to increase agreement. Statements that did not achieve consensus would be revised for a final Round 3.

Statement 1.R1

Statement: *CMD catheter placement should be performed by an appropriately trained clinician (e.g., neurosurgeon, neurosurgical trainee, or intensivist). A consultant/attending neurosurgeon should have overall responsibility for the procedure and ensure that support is available to manage any complications.*

Rationale for revision: The revised statement above was proposed based on feedback on the **Round 1 statement 2**. Feedback on the Round 1 statement indicated two main reasons for disagreement. First, the R1 statement was perceived as too restrictive by excluding appropriately trained trainees (e.g., residents, fellows) from performing the procedure, which many panelists viewed as impractical. Second, the phrase 'responsible for' was perceived as ambiguous, and it was unclear whether it meant direct performance or supervisory oversight. The proposed revision aims to increase consensus by explicitly including trainees, clarifying the distinction between performing the procedure and having overall responsibility, and assigning ultimate oversight to a senior neurosurgeon, which also addresses concerns about which specialty should lead.

Statement 2.R1

Statement: *While CMD is an inherently focal monitor, its data may be cautiously interpreted as representative of the hemispheric metabolic state in diffuse injury patterns, provided the catheter tip is placed in brain tissue which does not have a focal injury on neuroimaging.*

Rationale for revision: The revised statement above was proposed based on feedback on the **Round 1 statement 5**. Feedback on the Round 1 statement revealed two major issues contributing to disagreement. First, there was tension between CMD's nature as a focal monitor and the concept of generalizing its data to the 'whole brain'. Many panelists felt this was an overstatement, suggesting that data might represent a hemisphere or a larger region at best. Second, panelists noted that generalizability is highly dependent on the specific injury pathology

(e.g., TBI, SAH), pattern (focal vs. diffuse), and importantly, the specific analyte being measured; for instance, glucose measured in normal-appearing tissue may be more generalizable than a focal marker of cell membrane injury like glycerol. The wording of the original statement was criticized as vague, with calls to add specific conditions for generalization, such as placement in normal-appearing brain tissue, and to adopt a more cautious tone. The proposed revision aims to address these points by explicitly acknowledging the focal nature of CMD, narrowing the context to diffuse injuries, replacing 'whole brain' with the more conservative 'hemispheric', and adding a specific condition about catheter placement.

Statement 3.R1

Statement: *The use of two CMD catheters, which has been implemented safely in published series, can provide a more comprehensive insight than single catheter monitoring in cases with heterogeneous injury patterns.*

Rationale for revision: The revised statement above was proposed based on feedback on the **Round 1 statement 6**. Feedback indicated two main issues. First, the statement is compound, asking panelists to agree on both safety and value simultaneously. Second, the assertion that the practice 'is safe' was considered too absolute by many, who pointed to a lack of robust evidence and potential complications. Panelists also raised practical concerns about the complexity, cost, and logistical challenges of implementing multi-catheter monitoring. The proposed revision softens the language on safety to be more reflective of existing literature and reframes 'adds value' to the more cautious 'can provide more comprehensive insight,' which better aligns with the panel's sentiment that the practice is promising but not yet a proven standard. We also changed the wording from 'multiple' to 'two' catheters, to avoid generalizations.

Statement 4.R1

Statement: *In a prioritized approach to treating deranged metabolism with the intention of lowering the LPR, the following general sequence of priorities is suggested, while acknowledging that these parameters are often managed simultaneously depending on the clinical context: (1) ICP should be addressed with priority (target ≤ 20 mmHg); (2) then PbtO2 (target ≥ 15 mmHg); (3) and finally, brain glucose (target ≥ 1.0 mM); (4) Further interventions (e.g., targeting CPP) should be considered for refractory metabolic derangement as indicated by a persistently increased LPR (> 25).*

Rationale for revision: The revised statement above was proposed based on feedback on the **Round 1 statement 9**. Panelists were skeptical of a strict hierarchical approach, pointing to the parallel nature of how these targets are often managed. Softening our recommendation for a hierarchical approach by recognizing this reality may help to achieve agreement in the revised statement. Furthermore, panelists seemed to want an emphasis on the endpoint of LPR, so the modified version stresses the importance of monitoring LPR to gauge the effectiveness of these treatments on metabolic derangement. Lastly, panelists wanted recognition of other treatments such as CPP manipulation, so the modified statement mentions the utility of further interventions for refractory metabolic derangement.

Statement 5.R2

Statement: *The interpretation of an elevated lactate/pyruvate ratio (LPR) should consider the trend over time and the duration of the elevation, as an isolated value may be insufficient to change clinical management.*

Rationale: The new statement above was suggested by panelists during Round 1. It introduces the concepts of 'trend over time' and 'duration of elevation' as crucial factors for interpreting LPR, which were absent from other Round 1 statements that primarily focused on static thresholds without regard to the consistency of the derangement.

Statement 6.R1

Statement: *During neurological wake-up tests, worsening of CMD values may indicate an early warning of subsequent deterioration that can inform whether a wake-up test is aborted.*

Rationale for revision: The revised statement above was proposed based on feedback on the **Round 1 statement 17**. The feedback indicated that the original statement was too definitive for the current level of evidence. Panelists argued that the decision to perform a wake-up test is complex and depends on multiple factors, including ICP and clinical exam, not just isolated CMD values. The phrasing 'a trend of' was considered ambiguous. A key suggestion was to reframe the statement from avoiding the test to using CMD to monitor the patient's metabolic response during the test, allowing for modification or abortion of the procedure if CMD values worsen. The proposed revision incorporates this feedback by softening the language and shifting the focus to cautious monitoring during the test rather than avoidance.

Statement 7.R1

Statement: *When taken in a wider context including systemic physiology and cerebral oxygenation, raised LPR (> 25) can contribute to a decision to perform red blood cell transfusion (RBCT) in order to improve cerebral oxygenation.*

Rationale for revision: The revised statement above was proposed based on feedback on the **Round 1 statement 19**. Feedback indicates two primary reasons for disagreement and neutrality. First, panelists found the statement too prescriptive and lacking the necessary clinical context. They argued that the decision to transfuse is complex and must be integrated with a comprehensive assessment of other variables (e.g., ICP, CPP), the cause of anemia, and the risks of transfusion. Second, several experts questioned the utility of including the LPR value in decision criteria, noting that recent evidence from trials like TRAIN supports transfusion based on Hgb and PbtO2 thresholds irrespective of CMD findings. The proposed statement sidesteps many of these issues and gets to the core of CMD's role in indicating RBCT.

Statement 8.R1

Statement: *In TBI patients with a CMD pattern suggestive of ischemia (e.g., LPR > 25 and pyruvate < 70 μM), the initial response should be to evaluate and optimize cerebral perfusion pressure, brain tissue oxygenation, and cerebral autoregulation. If the ischemic pattern persists despite these measures, a short therapeutic trial of normobaric hyperoxia (e.g., a step increase*

in FiO2 for 2 hours) may be considered to assess for improvement in deranged cerebral metabolism.

Rationale for revision: The revised statement above was proposed based on feedback on the **Round 1 statement 21**. The primary objections from the panel were threefold. First, the recommendation is too strong for the current evidence base, which is largely observational. Second, it fails to prioritize the assessment and optimization of primary factors like cerebral perfusion pressure (CPP) and brain tissue oxygen (PbtO2) before escalating to hyperoxia. Third, the specific directive to use 100% FiO2 was contentious, with many panelists advocating for a more cautious approach of titrating FiO2 to a PaO2 target to avoid the potential harms of hyperoxemia. The proposed revision reframes the statement to address these concerns by establishing a clearer clinical workflow, softening the recommendation, and modifying the intervention to be more consistent with expert feedback. Our writing committee noted the sensitive nature of prescribing PaO2 thresholds and the specific method of inducing hyperoxia, leading us to simplify the statement and avoid an over-prescriptive statement.

Statement 9.R1

Statement: *In the traumatically injured brain, interstitial fluid glucose measured by microdialysis in tissue presumed to be uninjured has a positive correlation with plasma glucose. Consequently, brain glucose can be modulated by altering plasma glucose levels (e.g., via insulin or glucose administration). However, the predictability of this response should be determined empirically, as the relationship is influenced by factors such as catheter tip location, local cerebral blood flow, and metabolic rate.*

Rationale for revision: The revised statement above was proposed based on feedback on the **Round 1 statement 13**. The feedback indicates that the terms "controlled, predictable manner" and "positive linear relationship" are too strong and do not adequately capture the complexities observed in clinical practice. Panelists noted that the relationship is influenced by numerous factors, including probe location, local cerebral blood flow, and metabolic rate, which makes the response to interventions like glucose or insulin administration variable and less predictable than the statement implies. Panelists cited evidence suggesting that glucose levels in non-injured tissue may not be a reliable target due to different metabolic activity in more vulnerable brain regions[1] The proposed revision softens the language to reflect this uncertainty and complexity, acknowledging the general positive correlation while incorporating the key confounding factors raised by the panel.

References:

1. Hermanides J, Hong YT, Trivedi M, et al. Metabolic derangements are associated with impaired glucose delivery following traumatic brain injury. Brain. 2021;144(11):3492-3504. doi:10.1093/brain/awab255

Statement 10.R1

Statement: *Administration of intravenous dextrose may be used to raise plasma glucose up to 10 mM (180 mg/dL) in an effort to resolve neuroglycopenia.*

Rationale for revision: The revised statement above was proposed based on feedback on the **Round 1 statement 14**. The primary reasons for disagreement and neutrality were the prescriptive nature of '50% dextrose' and concerns about the strength of the evidence. Panelists noted that different concentrations of dextrose are used in practice (e.g., 30%) and that the evidence for the intervention's benefit is not considered robust by some. Panelists also suggested the additional functionality of such supplementation as a sort of 'challenge' to test the cerebral glucose responsiveness in cases of persistently low cerebral glucose. To address this, the revision removes the specific concentration, generalizing the statement to 'intravenous dextrose'. This change accommodates variations in clinical practice while retaining core guidance, which recent evidence has supported in SAH patients[1] (in addition to the TBI evidence we cited in Round 1).

References:

1. Kofler M, Lindner A, Rass V, et al. Liberalization of the Systemic Glucose Management is Associated with a Reduced Frequency of Neuroglucopenia in Subarachnoid Hemorrhage Patients: An Observational Cohort Study. Neurocrit Care. 2025;42(2):343-350. doi:10.1007/s12028-024-02126-8

Statement 11.R2

Statement: *In cases of neuroglycopenia, proactive metabolic support with alternative energy substrates, such as lactate or ketone bodies, may be considered.*

Rationale: The new statement above was suggested by panelists in Round 1. It introduces a new therapeutic strategy, as our original set of statements only focused on using dextrose to raise glucose levels (now covered in statement 10.R1).

Statement 12.R2

Statement: *Strategies to treat neuroglycopenia should include optimizing enteral nutrition.*

Rationale: The new statement above was suggested by panelists in R1. It proposes a therapeutic strategy for managing neuroglycopenia that is distinct from those of statements 10.R1 and 11.R2. The panelists provided supporting literature for this method of 'improving nutrition'.[1]

References:

1. Kofler M, Schiefecker AJ, Beer R, et al. Enteral nutrition increases interstitial brain glucose levels in poor-grade subarachnoid hemorrhage patients. J Cereb Blood Flow Metab. 2018;38(3):518-527. doi:10.1177/0271678X17700434

Statement 13.R2

Statement: *The ratio of cerebral to systemic (plasma) glucose is a useful parameter in the interpretation of CMD data.*

Rationale: The new statement above was suggested by panelists in Round 1. This concept[1] was not explicitly addressed in the original R1 statements.

References:

1. Kurtz P, Claassen J, Schmidt JM, et al. Reduced brain/serum glucose ratios predict cerebral metabolic distress and mortality after severe brain injury. Neurocrit Care. 2013;19(3):311-319. doi:10.1007/s12028-013-9919-x

Statement 14.R2

Statement: *Cerebral glycerol is a useful marker for monitoring secondary injury and cellular deterioration after traumatic brain injury (TBI).*

Rationale: The new statement above was suggested by the writing committee. It was felt that including a statement on glycerol would be beneficial, as it is considered a marker of cellular damage and complements another new question (15.R2) assessing the role of glutamate in secondary injury after TBI.

Statement 15.R2

Statement: *Cerebral glutamate is a useful marker for monitoring secondary injury and cellular deterioration after traumatic brain injury (TBI).*

Rationale: The new statement above was suggested by panelists in Round 1. They felt that the relevance of glutamate should be re-emphasized, providing nuance to a R1 survey statement: "The most reliable clinical CMD parameters are glucose and LPR, which provide more clinically actionable information than monitoring glutamate or glycerol." Panelists provided literature[1] supporting the relevance of glutamate in TBI.

References:

1. Hinzman JM, Wilson JA, Mazzeo AT, et al. Excitotoxicity and Metabolic Crisis Are Associated with Spreading Depolarizations in Severe Traumatic Brain Injury Patients. J Neurotrauma. 2016;33(13):1259-1267. doi:10.1089/neu.2015.4226

Statement 16.R2

Statement: *There is a relationship between glutamate and clinical outcome.*

Rationale: The new statement above was suggested by panelists in Round 1. Following on from the previous statement (15.R2), panelists felt it was important to specifically probe the perceived relationship between cerebral glutamate and clinical outcome. This statement aims to capture the panelists' literature- and anecdote-informed opinions on this distinct correlation.

Statement 17.R2

Statement: *Although evidence supporting CMD use in intracerebral hemorrhage (ICH) patients is still emerging, studies thus far are promising and highlight the importance of continued research in this area.*

Rationale: The new statement above serves as an opening for the following questions about the utility of CMD in monitoring and treating intracerebral hemorrhage (ICH). It allows panelists to vote separately on (1) the potential of CMD to monitor and guide ICH treatment,[1-5] and (2) the prevalence and strength of the evidence supporting this emerging practice.

References:

1. Kang J, Shah I, Shahrestani S, et al. Friedman's Gradient-Boosting Algorithm Predicts Lactate-Pyruvate Ratio Trends in Cases of Intracerebral Hemorrhages. World Neurosurg. 2024;187:e620-e628. doi:10.1016/j.wneu.2024.04.136
2. Shah I, Chen PM, Tran DKT, Chen JW. Cerebral microdialysis demonstrates improvements in brain metabolism with cerebrospinal fluid diversion in spontaneous intracerebral hemorrhage. Surg Neurol Int. 2023;14:395. Published 2023 Nov 10. doi:10.25259/SNI_679_2023
3. Rasulo F, Piva S, Park S, et al. The Association Between Peri-Hemorrhagic Metabolites and Cerebral Hemodynamics in Comatose Patients With Spontaneous Intracerebral Hemorrhage: An International Multicenter Pilot Study Analysis. Front Neurol. 2020;11:568536. Published 2020 Oct 26. doi:10.3389/fneur.2020.568536
4. Nikaina I, Paterakis K, Paraforos G, et al. Cerebral perfusion pressure, microdialysis biochemistry, and clinical outcome in patients with spontaneous intracerebral hematomas. J Crit Care. 2012;27(1):83-88. doi:10.1016/j.jcrc.2011.04.004
5. Lindner A, Rass V, Ianosi BA, et al. Individualized blood pressure targets in the postoperative care of patients with intracerebral hemorrhage. J Neurosurg. 2021;135(6):1656-1665. doi:10.3171/2020.9.JNS201024

Statement 18.R1

Statement: *CMD monitoring should be considered for ICH to monitor the particularly at-risk perihematomal zone in selected cases of severe injury when there is a concurrent indication for invasive cerebral monitoring (e.g., intracranial pressure) or when access is available at the time of neurosurgical procedure.*

Rationale for revision: The revised statement above was proposed based on feedback on the **Round 1 statement 1**. Since the sub-question regarding ICH failed to reach agreement, we are merging it with the main stem of the question in the Round 2 survey, which will allow for a more straight-forward, ICH-specific question. Feedback on the Round 1 statement indicated that low agreement with the ICH subquestion was due to the view that its use is primarily investigational and a desire for more specific selection criteria. Panelists suggested qualifying the indication by severity. The proposed revision incorporates these themes to build consensus. Further, we modified the statement to stress the utility of the CMD catheter when placed in the at-risk perihematomal zone.[1-4]

References:

1. Kang J, Shah I, Shahrestani S, et al. Friedman's Gradient-Boosting Algorithm Predicts Lactate-Pyruvate Ratio Trends in Cases of Intracerebral Hemorrhages. World Neurosurg. 2024;187:e620-e628. doi:10.1016/j.wneu.2024.04.136
2. Shah I, Chen PM, Tran DKT, Chen JW. Cerebral microdialysis demonstrates improvements in brain metabolism with cerebrospinal fluid diversion in spontaneous intracerebral hemorrhage. Surg Neurol Int. 2023;14:395. Published 2023 Nov 10. doi:10.25259/SNI_679_2023
3. Rasulo F, Piva S, Park S, et al. The Association Between Peri-Hemorrhagic Metabolites and Cerebral Hemodynamics in Comatose Patients With Spontaneous Intracerebral

Hemorrhage: An International Multicenter Pilot Study Analysis. Front Neurol. 2020;11:568536. Published 2020 Oct 26. doi:10.3389/fneur.2020.568536
4. Nikaina I, Paterakis K, Paraforos G, et al. Cerebral perfusion pressure, microdialysis biochemistry, and clinical outcome in patients with spontaneous intracerebral hematomas. J Crit Care. 2012;27(1):83-88. doi:10.1016/j.jcrc.2011.04.004

Statement 19.R1

Statement: *CMD is capable of indicating metabolic changes associated with the development of cerebral edema in the perihematomal zone of ICH, particularly when the catheter tip is placed in this region.*

Rationale for revision: The revised statement above was proposed based on feedback on the **Round 1 statement 8**. Feedback indicates two primary reasons for the disagreement with the ICH sub-statement. First, it was thought that there was insufficient evidence to conclude predictive ability of CMD in predicting edema in the perihematomal zone of ICH. Second, feedback indicated that the utility of CMD is critically dependent on the precise placement of the catheter in the perihematomal zone.[1-4] The proposed revision reframes the statement to focus on monitoring 'metabolic changes associated with' edema rather than predicting edema itself and explicitly adds the critical qualifier about catheter location to increase the likelihood of achieving consensus. For the modified statement, we have merged the ICH sub-statement into the main-stem for more tailored delivery.

References:

1. Kang J, Shah I, Shahrestani S, et al. Friedman's Gradient-Boosting Algorithm Predicts Lactate-Pyruvate Ratio Trends in Cases of Intracerebral Hemorrhages. World Neurosurg. 2024;187:e620-e628. doi:10.1016/j.wneu.2024.04.136
2. Shah I, Chen PM, Tran DKT, Chen JW. Cerebral microdialysis demonstrates improvements in brain metabolism with cerebrospinal fluid diversion in spontaneous intracerebral hemorrhage. Surg Neurol Int. 2023;14:395. Published 2023 Nov 10. doi:10.25259/SNI_679_2023
3. Rasulo F, Piva S, Park S, et al. The Association Between Peri-Hemorrhagic Metabolites and Cerebral Hemodynamics in Comatose Patients With Spontaneous Intracerebral Hemorrhage: An International Multicenter Pilot Study Analysis. Front Neurol. 2020;11:568536. Published 2020 Oct 26. doi:10.3389/fneur.2020.568536
4. Nikaina I, Paterakis K, Paraforos G, et al. Cerebral perfusion pressure, microdialysis biochemistry, and clinical outcome in patients with spontaneous intracerebral hematomas. J Crit Care. 2012;27(1):83-88. doi:10.1016/j.jcrc.2011.04.004

Statement 20.R1

Statement: *In patients undergoing ICH resection, CMD may be considered as an adjunct to standard monitoring to assess the metabolic response to surgery. With the catheter tip placed in the perihematomal zone, worsening or non-recovering metabolic derangements despite resection can help inform the need for further investigation or intervention (e.g., EVD placement).*

Rationale for revision: Panelists felt the **Round 1 statement 20** was too prescriptive and too strong for the existing level of evidence, primarily from smaller studies. There was also a need

for greater clarity regarding the clinical application, specifically emphasizing its role as an adjunct to standard monitoring (not a replacement) and the importance of probe placement in the perihematomal tissue to ensure relevant data is collected. The proposed revision aims to soften the language and add necessary clinical context regarding probe location and its adjunctive role.

Statement 21-25.R2

Statement: The following items should be considered core reporting items: (*21.R2) reference values used for each monitored CMD parameter (e.g.: 0.2 mM, 0.8 mM, or 1.0 mM for brain glucose; 25 or 40 for LPR; etc.); (22.R2) depth of CMD catheter tip; (23.R2) duration of adequate CMD system (i.e., machine, catheter, etc.) functioning and total duration of CMD monitoring; (24.R2) if available, hourly plasma glucose records in addition to cerebral glucose records; (25.R2) if intravenous glucose supplementation is provided, the rate (mmol/hour) and concentration of the supplementation alongside the set of, if available, associated plasma and cerebral glucose records.*

Rationale: The new statements above represent potential core reporting items suggested by panelists during Round 1. Since all originally proposed core reporting items achieved ≥75% agreement, this section seeks to establish consensus on these additional items to ensure the meaningfulness of data and to aid in the communication of CMD results.

Statement 26.R1

Statement: *Although center-dependent, the lack of automated streaming of CMD data to electronic medical records poses a barrier to the efficient clinical implementation of CMD monitoring.*

Rationale for revision: The revised statement above was proposed based on feedback on the **Round 1 statement 25.6**. Feedback indicates that the absolute wording 'lack of ability' is problematic, as some centers have developed solutions for EMR integration. Panelists suggested that this is not a universal barrier. Modifying the text to reflect the 'difficulty' rather than a complete 'lack of ability' acknowledges the challenge while accommodating the experiences of centers that have overcome it. This revised statement also drops the word 'largest' when referring to the barrier.

Statement 27.R2

Statement: *Although region and center-dependent, lack of approval of CMD for routine clinical use by some regulatory agencies presents a barrier to the clinical implementation of CMD monitoring.*

Rationale: The new statement above was suggested by panelists in Round 1. It addresses a key barrier to implementation: the lack of approval for routine clinical use by some regulatory agencies, which can be a significant region- and center-dependent obstacle to wider clinical adoption.

Round 3 survey

Round 3 was the final round, presenting refined versions of statements that did not achieve consensus in Round 2. Panelists were informed that this was the last round and that statements would not be revised further regardless of outcome but that feedback and comments were nonetheless welcome.

Opening questions

1. Full name — as it should appear on any publication
2. Preferred email address

Consensus statements

Response scale: Strongly Agree | Agree | Neutral | Disagree | Strongly Disagree | Decline to Respond

Although no further revisions would occur, panelists were still welcome to provide qualitative feedback for the record.

Statement 1.R1

Statement: *In a prioritized approach to treating deranged metabolism with the intention of lowering the LPR, the following general sequence of priorities is suggested, while acknowledging that these parameters are often managed simultaneously depending on the clinical context: (1) ICP should be addressed with priority (target ≤ 20 mmHg); (2) then PbtO2 (target ≥ 15 mmHg); (3) and finally, brain glucose should be monitored, with investigation and management of systemic causes for levels below 1.0 mM; (4) Further interventions (e.g., targeting CPP) should be considered for refractory metabolic derangement as indicated by a persistently increased LPR (> 25).*

Rationale for revision: The revised statement above was proposed based on feedback on the **Round 2 statement 4.R1**. While three of the four sub-questions achieved consensus in Round 2, the third sub-question regarding brain glucose failed to reach the consensus threshold. The primary concern was the actionability of targeting brain glucose, with panelists expressing skepticism about whether systemic glucose administration can reliably increase brain glucose levels, especially during metabolic crisis. The proposed revision shifts the focus from actively 'targeting' a specific brain glucose value to monitoring for critically low levels and managing potential systemic causes. This addresses concerns about feasibility and mechanism while maintaining the clinical relevance of monitoring this parameter. Additionally, although there was significant feedback challenging the hierarchical approach and suggesting CPP should be managed earlier and more prominently, these changes were not incorporated because the relevant sub-questions achieved consensus per the decision rules. It should be noted that this prioritized sequence applies to patients undergoing multimodal monitoring that includes ICP, PbtO2, and CMD; however, some centers may use microdialysis without oxygen monitoring, and clinical judgment should guide the application of these priorities in such contexts.

Statement 2.R1

Statement: *Worsening CMD values may indicate tissue vulnerability and can help inform the decision to defer, modify, or abort a neurological wake-up test or a trial of sedation lightening.*

Rationale for revision: The revised statement above was proposed based on feedback on the **Round 2 statement 6.R1**. The statement did not achieve the consensus threshold. The primary concern raised in the qualitative feedback was the slow temporal resolution of CMD, which panelists felt was inadequate for making real-time decisions to abort a brief neurological wake-up test. Feedback suggested that CMD is more applicable for monitoring during longer trials of sedation lightening or for making an upfront decision to defer a test based on pre-existing metabolic instability. Additionally, panelists noted that CMD data should be considered in conjunction with other clinical parameters, such as ICP and the clinical exam, rather than in isolation. The proposed revision broadens the context from just 'wake-up tests' to include 'sedation lightening' and expands the potential actions from 'aborted' to 'defer, modify, or abort' to better reflect these practical considerations.

Statement 3.R1

Statement: *In TBI patients with a CMD pattern suggestive of ischemia (e.g., LPR > 25 and pyruvate < 70 µM), the initial response should be to evaluate and optimize cerebral perfusion pressure, brain tissue oxygenation, cerebral autoregulation, and brain glucose levels. If the ischemic pattern persists despite these measures, a short (e.g., up to 2 hours) trial of normobaric hyperoxia may be considered as a diagnostic challenge to assess the responsiveness of the metabolic derangement.*

Rationale for revision: The revised statement above was proposed based on feedback on the **Round 2 statement 8.R1**. The statement did not achieve the consensus threshold. The primary objections stem from a fundamental disagreement regarding the therapeutic benefit and safety of normobaric hyperoxia, with some panelists citing evidence that it may not improve outcomes and could be harmful. A second major point of contention was the omission of brain glucose management from the list of initial optimization parameters. To address these concerns and build consensus, the proposed revision incorporates the optimization of brain glucose levels. Furthermore, it reframes the hyperoxia intervention from a 'therapeutic trial' to a 'diagnostic challenge,' which may be more acceptable to panelists who question its therapeutic efficacy but acknowledge its potential use in assessing metabolic responsiveness.

Statement 4.R2

Statement: *The administration of alternative energy substrates, such as lactate or ketone bodies, to manage neuroglycopenia is an investigational strategy that requires further study before it can be recommended for clinical use.*

Rationale for revision: The revised statement above was proposed based on feedback on the **Round 2 statement 11.R2**. The statement did not achieve the consensus threshold. The dominant feedback from panelists is that this strategy is experimental, lacks sufficient evidence, and is not ready for routine clinical use. Concerns were also raised about practical issues like availability and administration, as well as potential systemic repercussions. One panelist cited a review suggesting alternative substrates may not fully substitute for glucose. To address these

widespread concerns, the proposed revision reframes the statement to explicitly classify this approach as investigational and in need of further research, rather than a therapeutic option that 'may be considered' in current practice. This change directly incorporates the panel's primary objection and aims to build consensus by acknowledging the current state of the evidence.

Statement 5.R2

Statement: *Systemic (plasma) glucose levels should be considered when interpreting cerebral microdialysis glucose data.*

Rationale for revision: The revised statement above was proposed based on feedback on the **Round 2 statement 13.R2**. The statement did not reach consensus. The qualitative feedback indicates that while the concept is relevant, the specific focus on the 'ratio' as a 'useful parameter' is contentious. Panelists raised concerns about the lack of evidence, unclear clinical utility, and absence of specific guidance on how to interpret the ratio. One panelist suggested that focusing on the ratio might obscure the importance of the absolute cerebral glucose value. A simpler, less prescriptive statement was suggested that captures the underlying principle without mandating the use of a specific 'ratio', which is likely to achieve higher consensus.

Statement 6.R2

Statement: *Elevated cerebral glycerol is an indicator of cell death and damage from secondary injury after traumatic brain injury (TBI).*

Rationale for revision: The revised statement above was proposed based on feedback on the **Round 2 statement 14.R2**. The statement did not reach consensus, with significant disagreement centered on the terms 'useful' and 'monitoring'. Feedback indicates that many panelists do not find glycerol clinically useful for guiding management due to its perceived unreliability and erratic nature. Furthermore, there is a strong sentiment that glycerol is a late marker, indicating damage that has already occurred, rather than a tool for monitoring an ongoing secondary injury. The proposed revision reframes the statement to be more factual, removing the subjective term 'useful' and changing the role from active 'monitoring' to a more passive 'indicator' of cell death and damage, which aligns with the expert feedback.

Statement 7.R2

Statement: *An increase in cerebral glutamate is indicative of secondary injury, such as from excitotoxicity, after traumatic brain injury (TBI).*

Rationale for revision: The revised statement above was proposed based on feedback on the **Round 2 statement 15.R2**. The statement did not reach consensus. The qualitative feedback indicates that the primary source of disagreement is the word 'useful,' which panelists felt implies a level of clinical actionability that they have not experienced in practice. Panelists commented that glutamate has been 'less reliable' or that they have 'not found CMD glutamate useful in clinical management.' A constructive suggestion was offered to rephrase the statement to be more factual, focusing on what an increase in glutamate indicates rather than its clinical utility. The proposed revision adopts this suggestion by stating that an increase in glutamate 'is indicative of' secondary injury, with excitotoxicity noted as one key mechanism, which is a more objective statement likely to achieve higher consensus.

Statement 8.R2

Statement: *Increased cerebral glutamate is associated with poor clinical outcome.*

Rationale for revision: The revised statement above was proposed based on feedback on the **Round 2 statement 16.R2**. The statement did not reach consensus. Feedback indicated that the statement is too ambiguous, as it does not specify the direction of the relationship. One panelist explicitly stated they would agree that 'increased glutamate is associated with poor outcome.' Other panelists expressed uncertainty about the strength and reliability of the evidence. The proposed revision directly addresses the ambiguity by specifying the direction of the relationship, creating a more precise statement for the panel to evaluate.

Statement 9.R1

Statement: *In patients undergoing ICH resection, CMD may be considered as an adjunct to standard monitoring to assess the metabolic response to surgery. With the catheter tip placed in the perihematomal zone, worsening or non-recovering metabolic derangements despite resection may signal an evolving secondary injury, prompting further investigation to identify potential causes.*

Rationale for revision: The revised statement above was proposed based on feedback on the **Round 2 statement 20.R1**. The statement did not achieve the consensus threshold. The primary concern raised in the qualitative feedback was that the statement was too prescriptive, particularly the phrase 'can help inform the need for further investigation or intervention'. Panelists felt this was too strong for the current level of evidence and expressed reservations about using CMD to directly guide clinical decisions. There were also comments on the practical difficulty of probe placement and the need for cautious interpretation of post-surgical metabolic changes. The proposed revision aims to increase consensus by softening the language, removing the direct link to 'intervention' and the specific example of EVD placement. It reframes CMD's role as a tool that may signal a potential problem requiring further diagnostic workup, rather than one that dictates a specific therapeutic response.

Statement 10.R2

Statement: *If available, plasma glucose records should be considered core reporting items in conjunction with cerebral glucose records.*

Rationale for revision: The revised statement above was proposed based on feedback on the **Round 2 statement 24.R2**. This statement did not reach the consensus threshold. While specific feedback was limited, the term 'hourly' may be perceived as overly prescriptive, as plasma glucose monitoring practices can vary. Removing this specific time frequency makes the statement more flexible and may increase agreement by accommodating different institutional protocols, while still capturing the importance of reporting contemporaneous plasma glucose data when available.

Supplement 2: quantitative statement results

Legend for Responses:

SA = Strongly Agree | **A** = Agree | **N** = Neutral | **D** = Disagree | **SD** = Strongly Disagree

N/A = Not Applicable / Decline to Respond

Agreement = Percentage of non-abstaining panelists voting Strongly Agree or Agree

Round 1 results

39 panelists participated in this round.

Question 1

Main Statement Stem: *Cerebral microdialysis (CMD) monitoring should be considered for the following condition(s) when there is a concurrent indication for invasive cerebral monitoring (e.g., intracranial pressure) or when access is available at the time of neurosurgical procedure.*

Sub-question: *traumatic brain injury (TBI)*

91.9% (34/37) — SA: 24, A: 10, N: 2, D: 0, SD: 1, N/A: 0

Sub-question: *subarachnoid hemorrhage (SAH)*

88.9% (32/36) — SA: 19, A: 13, N: 2, D: 1, SD: 1, N/A: 1

Sub-question: *intracerebral hemorrhage (ICH)*

51.5% (17/33) — SA: 4, A: 13, N: 12, D: 3, SD: 1, N/A: 4

Question 2

Statement: *Consultant or attending neurosurgeons or appropriately trained intensivists should be responsible for CMD catheter placement in order to avoid adverse events and deal with any complications.*

70.3% (26/37) — SA: 22, A: 4, N: 4, D: 6, SD: 1, N/A: 0

Question 3

Statement: *Dependent on local availability and expertise, CMD catheters can be placed by twist drill hole, transcranial bolt, or during open craniotomy or craniectomy, with each of these procedures being safe and effective.*

97.2% (35/36) — SA: 29, A: 6, N: 0, D: 0, SD: 1, N/A: 1

Question 4

Statement: *The first hour of microdialysate should not be used to make clinical decisions due to the confounders of catheter insertion and pump flushing.*

100.0% (37/37) — SA: 32, A: 5, N: 0, D: 0, SD: 0, N/A: 0

Question 5

Statement: *Focally recorded CMD data may be generalizable to the whole brain depending on the location of the catheter with respect to any focal lesions and the pattern of brain injury.*

70.3% (26/37) — SA: 10, A: 16, N: 3, D: 8, SD: 0, N/A: 0

Question 6

Statement: *Use of multiple microdialysis catheters is safe and adds value with more robust insight than single catheter monitoring in cases where several patterns of injury occur in the same patient.*

67.6% (25/37) — SA: 12, A: 13, N: 6, D: 6, SD: 0, N/A: 0

Question 7

Statement: *Integration of CMD data with multimodal physiological signals is essential for holistic clinical decision making.*

89.2% (33/37) — SA: 28, A: 5, N: 3, D: 0, SD: 1, N/A: 0

Question 8

Main Statement Stem: *CMD can provide early indication of impending deterioration and secondary injury such as*

Sub-question: *intracranial hypertension in TBI*

89.2% (33/37) — SA: 18, A: 15, N: 2, D: 1, SD: 1, N/A: 0

Sub-question: *symptomatic delayed ischemia in SAH*

91.7% (33/36) — SA: 19, A: 14, N: 3, D: 0, SD: 0, N/A: 1

Sub-question: *cerebral edema in the perihematomal zone of ICH (intracerebral hemorrhage)*

63.3% (19/30) — SA: 6, A: 13, N: 10, D: 1, SD: 0, N/A: 7

Question 9

Main Statement Stem: *In a hierarchical approach to treating deranged metabolism using a CMD-inclusive multimodal monitoring scheme:*

Sub-question: *1. ICP should be addressed with priority (target ≤ 20 mmHg),*

83.8% (31/37) — SA: 24, A: 7, N: 4, D: 1, SD: 1, N/A: 0

Sub-question: *2. then PbtO2 (target ≥ 15 mmHg),*

91.9% (34/37) — SA: 21, A: 13, N: 1, D: 1, SD: 1, N/A: 0

Sub-question: *3. and finally, brain glucose (target ≥ 1.0 mM).*

70.3% (26/37) — SA: 19, A: 7, N: 8, D: 2, SD: 1, N/A: 0

Question 10

Main Statement Stem: *For those patients with abnormally high LPR (> 25) detected by CMD despite other multimodal parameters having been normalized in line with locally determined treatment protocols:*

Sub-question: *Ischemia is indicated by concurrently insufficient substrate (e.g., brain glucose < 1.0 mM or pyruvate < 70 µM).*

86.1% (31/36) — SA: 16, A: 15, N: 2, D: 3, SD: 0, N/A: 1

Sub-question: *Clinical mitochondrial dysfunction is indicated by concurrently sufficient substrate (e.g., brain glucose ≥ 1.0 mM and pyruvate ≥ 70 µM).*

88.9% (32/36) — SA: 13, A: 19, N: 2, D: 2, SD: 0, N/A: 1

Question 11

Statement: *The most reliable clinical CMD parameters are glucose and LPR, which provide more clinically actionable information than monitoring glutamate or glycerol.*

100.0% (37/37) — SA: 27, A: 10, N: 0, D: 0, SD: 0, N/A: 0

Question 12

Statement: *Absolute lactate and pyruvate concentrations, alongside glucose, should be considered when interpreting heightened LPR, as this can indicate if the abnormality relates to a failure of metabolic substrate delivery.*

94.6% (35/37) — SA: 22, A: 13, N: 1, D: 1, SD: 0, N/A: 0

Question 13

Statement: *Brain glucose as recorded from non-injured tissue can be manipulated in a controlled, predictable manner in the traumatically injured brain (e.g., by insulin infusion or glucose administration), as it holds a positive linear relationship with plasma glucose.*

70.3% (26/37) — SA: 11, A: 15, N: 4, D: 7, SD: 0, N/A: 0

Question 14

Statement: *Administration of 50% dextrose may be used to raise plasma glucose up to 10 mM (180 mg/dL) in an effort to resolve neuroglycopenia (brain glucose < 1.0 mM).*

73.5% (25/34) — SA: 11, A: 14, N: 5, D: 4, SD: 0, N/A: 3

Question 15

Statement: *Despite the association of increased cerebral glutamate with brain glucose > 5.0 mM, there is insufficient evidence to define a critical upper limit for brain glucose.*

91.2% (31/34) — SA: 21, A: 10, N: 3, D: 0, SD: 0, N/A: 3

Question 16

Statement: *Although a CPP of 60-70 mmHg is recommended within Brain Trauma Foundation Guidelines, metabolic state as indicated by CMD has the potential to inform individualization of CPP targets.*

86.5% (32/37) — SA: 18, A: 14, N: 4, D: 1, SD: 0, N/A: 0

Question 17

Statement: *For optimal safety during episodes of vulnerability indicated by CMD, neurological wake-up tests may not be advisable in patients with a trend of insufficient cerebral glucose (< 1.0 mM) or high LPR (> 25).*

71.4% (25/35) — SA: 16, A: 9, N: 2, D: 8, SD: 0, N/A: 2

Question 18

Statement: *Disturbance of the brain's metabolic state as indicated by CMD can guide de-escalation of therapy because it demonstrates a remaining vulnerability to physiological perturbations.*

82.9% (29/35) — SA: 16, A: 13, N: 5, D: 1, SD: 0, N/A: 2

Question 19

Statement: *In cases of concurrent Hgb < 9.0 g/dL with PbtO2 < 20 mmHg, the triggering of red blood cell transfusion should be made more likely if LPR > 25 considering the inverse relationship of anemic Hgb and LPR.*

73.0% (27/37) — SA: 11, A: 16, N: 8, D: 2, SD: 0, N/A: 0

Question 20

Statement: *CMD may be used adjunctly to assess recovery following ICH resection and indicate the need for further interventions (e.g., EVD placement) in cases of non-responding or worsening condition despite resection.*

56.7% (17/30) — SA: 7, A: 10, N: 9, D: 3, SD: 1, N/A: 7

Question 21

Statement: *TBI presenting with concomitant lactate > 3.5 mM and pyruvate < 120 μM indicates the utility of short (e.g., 2 hours) normobaric hyperoxia (100% FiO2, achieving pO2 > 12 kPa) as a therapeutic trial to mitigate deranged cerebral metabolism.*

47.2% (17/36) — SA: 5, A: 12, N: 11, D: 5, SD: 3, N/A: 1

Question 22

Statement: *Hourly CMD sampling is sufficient to add clinical benefit.*

81.1% (30/37) — SA: 18, A: 12, N: 3, D: 3, SD: 1, N/A: 0

Question 23

Statement: *Higher frequency sampling (sub-hourly) or continuous monitoring may increase the utility of CMD as online monitoring systems are developed.*

81.1% (30/37) — SA: 16, A: 14, N: 4, D: 3, SD: 0, N/A: 0

Question 24

Main Statement Stem: *Core reporting items include:*

Sub-question: *mechanism used for catheter placement (e.g., twist drill hole, transcranial bolt, during open craniotomy or craniectomy)*

97.3% (36/37) — SA: 30, A: 6, N: 1, D: 0, SD: 0, N/A: 0

Sub-question: *catheter type*

100.0% (37/37) — SA: 32, A: 5, N: 0, D: 0, SD: 0, N/A: 0

Sub-question: *probe membrane length*

94.6% (35/37) — SA: 29, A: 6, N: 1, D: 1, SD: 0, N/A: 0

Sub-question: *lesion-relative catheter location confirmed by imaging*

100.0% (37/37) — SA: 32, A: 5, N: 0, D: 0, SD: 0, N/A: 0

Sub-question: *perfusion fluid composition*

100.0% (37/37) — SA: 30, A: 7, N: 0, D: 0, SD: 0, N/A: 0

Sub-question: *perfusion flow rate*

100.0% (37/37) — SA: 32, A: 5, N: 0, D: 0, SD: 0, N/A: 0

Sub-question: *handling of early samples (e.g., if the first hour or two of samples were discarded)*

97.3% (36/37) — SA: 25, A: 11, N: 1, D: 0, SD: 0, N/A: 0

Sub-question: *handling of values outside of analyzer limits of detection, missing values, and artifacts*

97.3% (36/37) — SA: 27, A: 9, N: 1, D: 0, SD: 0, N/A: 0

Sub-question: *other concurrent monitoring modalities*

100.0% (36/36) — SA: 30, A: 6, N: 0, D: 0, SD: 0, N/A: 1

Sub-question: *method of aligning multimodal data streams with different sampling frequencies (e.g., windowed averaging, nearest-neighbor sampling, weighted averaging)*

88.6% (31/35) — SA: 19, A: 12, N: 4, D: 0, SD: 0, N/A: 2

Sub-question: *neuroprotective procedures during monitoring*

89.2% (33/37) — SA: 24, A: 9, N: 3, D: 1, SD: 0, N/A: 0

Sub-question: *time from ictus*

100.0% (37/37) — SA: 28, A: 9, N: 0, D: 0, SD: 0, N/A: 0

Sub-question: *time from operation (if applicable)*

97.3% (36/37) — SA: 25, A: 11, N: 1, D: 0, SD: 0, N/A: 0

Sub-question: *complications associated with probe placement or removal*

97.3% (36/37) — SA: 28, A: 8, N: 1, D: 0, SD: 0, N/A: 0

Question 25

Main Statement Stem: *Currently, the largest barriers to clinical implementation of CMD monitoring are center-dependent, but generally include:*

Sub-question: *financial constraints*

91.9% (34/37) — SA: 20, A: 14, N: 3, D: 0, SD: 0, N/A: 0

Sub-question: *training of staff on operating procedures*

81.1% (30/37) — SA: 17, A: 13, N: 4, D: 3, SD: 0, N/A: 0

Sub-question: *lack of buy-in for CMD catheter placement*

83.3% (30/36) — SA: 19, A: 11, N: 5, D: 1, SD: 0, N/A: 1

Sub-question: *the time demand on staff from the handling of CMD samples (usually hourly)*

89.2% (33/37) — SA: 20, A: 13, N: 3, D: 0, SD: 1, N/A: 0

Sub-question: *integration of CMD trends with other multimodal device outputs*

78.4% (29/37) — SA: 17, A: 12, N: 2, D: 6, SD: 0, N/A: 0

Sub-question: *lack of ability to stream CMD data to electronic medical records*

62.2% (23/37) — SA: 13, A: 10, N: 7, D: 6, SD: 1, N/A: 0

Sub-question: *difficulty of interpreting high dimensional data*

75.7% (28/37) — SA: 18, A: 10, N: 3, D: 4, SD: 2, N/A: 0

Sub-question: *a limiting preconception of CMD being exclusive to research applications*

83.8% (31/37) — SA: 24, A: 7, N: 4, D: 1, SD: 1, N/A: 0

Sub-question: *a limiting preconception that CMD usage does not affect outcomes*

86.5% (32/37) — SA: 26, A: 6, N: 4, D: 1, SD: 0, N/A: 0

Round 2 results

35 panelists participated in this round.

Question 1.R1

Statement: *CMD catheter placement should be performed by an appropriately trained clinician (e.g., neurosurgeon, neurosurgical trainee, or intensivist). A consultant/attending neurosurgeon should have overall responsibility for the procedure and ensure that support is available to manage any complications.*

91.4% (32/35) — SA: 30, A: 2, N: 1, D: 1, SD: 1, N/A: 0

Question 2.R1

Statement: *While CMD is an inherently focal monitor, its data may be cautiously interpreted as representative of the hemispheric metabolic state in diffuse injury patterns, provided the catheter tip is placed in brain tissue which does not have a focal injury on neuroimaging.*

94.3% (33/35) — SA: 13, A: 20, N: 1, D: 1, SD: 0, N/A: 0

Question 3.R1

Statement: *The use of two CMD catheters, which has been implemented safely in published series, can provide a more comprehensive insight than single catheter monitoring in cases with heterogeneous injury patterns.*

82.9% (29/35) — SA: 11, A: 18, N: 3, D: 3, SD: 0, N/A: 0

Question 4.R1

Main Statement Stem: *In a prioritized approach to treating deranged metabolism with the intention of lowering the LPR, the following general sequence of priorities is suggested, while acknowledging that these parameters are often managed simultaneously depending on the clinical context:*

Sub-question: *1. ICP should be addressed with priority (target ≤ 20 mmHg),*

94.3% (33/35) — SA: 19, A: 14, N: 0, D: 2, SD: 0, N/A: 0

Sub-question: *2. then PbtO2 (target ≥ 15 mmHg),*

91.4% (32/35) — SA: 17, A: 15, N: 1, D: 2, SD: 0, N/A: 0

Sub-question: *3. and finally, brain glucose (target ≥ 1.0 mM).*

74.3% (26/35) — SA: 12, A: 14, N: 5, D: 4, SD: 0, N/A: 0

Sub-question: *4. Further interventions (e.g., targeting CPP) should be considered for refractory metabolic derangement as indicated by a persistently increased LPR (> 25).*

88.2% (30/34) — SA: 12, A: 18, N: 2, D: 2, SD: 0, N/A: 1

Question 5.R2

Statement: *The interpretation of an elevated lactate/pyruvate ratio (LPR) should consider the trend over time and the duration of the elevation, as an isolated value may be insufficient to change clinical management.*

97.1% (34/35) — SA: 26, A: 8, N: 0, D: 1, SD: 0, N/A: 0

Question 6.R1

Statement: *During neurological wake-up tests, worsening of CMD values may indicate an early warning of subsequent deterioration that can inform whether a wake-up test is aborted.*

71.9% (23/32) — SA: 9, A: 14, N: 7, D: 1, SD: 1, N/A: 3

Question 7.R1

Statement: *When taken in a wider context including systemic physiology and cerebral oxygenation, raised LPR (> 25) can contribute to a decision to perform red blood cell transfusion (RBCT) in order to improve cerebral oxygenation.*

85.3% (29/34) — SA: 9, A: 20, N: 3, D: 2, SD: 0, N/A: 1

Question 8.R1

Statement: *In TBI patients with a CMD pattern suggestive of ischemia (e.g., LPR > 25 and pyruvate < 70 µM), the initial response should be to evaluate and optimize cerebral perfusion pressure, brain tissue oxygenation, and cerebral autoregulation. If the ischemic pattern persists*

despite these measures, a short therapeutic trial of normobaric hyperoxia (e.g., a step increase in FiO2 for 2 hours) may be considered to assess for improvement in deranged cerebral metabolism.

71.4% (25/35) — SA: 6, A: 19, N: 4, D: 5, SD: 1, N/A: 0

Question 9.R1

Statement: *In the traumatically injured brain, interstitial fluid glucose measured by microdialysis in tissue presumed to be uninjured has a positive correlation with plasma glucose. Consequently, brain glucose can be modulated by altering plasma glucose levels (e.g., via insulin or glucose administration). However, the predictability of this response should be determined empirically, as the relationship is influenced by factors such as catheter tip location, local cerebral blood flow, and metabolic rate.*

88.6% (31/35) — SA: 17, A: 14, N: 1, D: 3, SD: 0, N/A: 0

Question 10.R1

Statement: *Administration of intravenous dextrose may be used to raise plasma glucose up to 10 mM (180 mg/dL) in an effort to resolve neuroglycopenia.*

75.8% (25/33) — SA: 10, A: 15, N: 5, D: 3, SD: 0, N/A: 2

Question 11.R2

Statement: *In cases of neuroglycopenia, proactive metabolic support with alternative energy substrates, such as lactate or ketone bodies, may be considered.*

34.4% (11/32) — SA: 3, A: 8, N: 14, D: 6, SD: 1, N/A: 3

Question 12.R2

Statement: *Strategies to treat neuroglycopenia should include optimizing enteral nutrition.*

82.4% (28/34) — SA: 14, A: 14, N: 5, D: 1, SD: 0, N/A: 1

Question 13.R2

Statement: *The ratio of cerebral to systemic (plasma) glucose is a useful parameter in the interpretation of CMD data.*

60.0% (21/35) — SA: 9, A: 12, N: 13, D: 1, SD: 0, N/A: 0

Question 14.R2

Statement: *Cerebral glycerol is a useful marker for monitoring secondary injury and cellular deterioration after traumatic brain injury (TBI).*

54.3% (19/35) — SA: 2, A: 17, N: 8, D: 8, SD: 0, N/A: 0

Question 15.R2

Statement: *Cerebral glutamate is a useful marker for monitoring secondary injury and cellular deterioration after traumatic brain injury (TBI).*

57.1% (20/35) — SA: 7, A: 13, N: 8, D: 7, SD: 0, N/A: 0

Question 16.R2

Statement: *There is a relationship between glutamate and clinical outcome.*

45.7% (16/35) — SA: 5, A: 11, N: 13, D: 6, SD: 0, N/A: 0

Question 17.R2

Statement: *Although evidence supporting CMD use in intracerebral hemorrhage (ICH) patients is still emerging, studies thus far are promising and highlight the importance of continued research in this area.*

88.6% (31/35) — SA: 13, A: 18, N: 4, D: 0, SD: 0, N/A: 0

Question 18.R1

Statement: *CMD monitoring should be considered for ICH to monitor the particularly at-risk perihematomal zone in selected cases of severe injury when there is a concurrent indication for invasive cerebral monitoring (e.g., intracranial pressure) or when access is available at the time of neurosurgical procedure.*

85.3% (29/34) — SA: 7, A: 22, N: 4, D: 1, SD: 0, N/A: 1

Question 19.R1

Statement: *CMD is capable of indicating metabolic changes associated with the development of cerebral edema in the perihematomal zone of ICH, particularly when the catheter tip is placed in this region.*

77.1% (27/35) — SA: 8, A: 19, N: 8, D: 0, SD: 0, N/A: 0

Question 20.R1

Statement: *In patients undergoing ICH resection, CMD may be considered as an adjunct to standard monitoring to assess the metabolic response to surgery. With the catheter tip placed in the perihematomal zone, worsening or non-recovering metabolic derangements despite resection can help inform the need for further investigation or intervention (e.g., EVD placement).*

70.6% (24/34) — SA: 7, A: 17, N: 9, D: 1, SD: 0, N/A: 1

Question 21-25.R2

Main Statement Stem: *The following items should be considered core reporting items:*

Sub-question: *21.R2. reference values used for each monitored CMD parameter (e.g.: 0.2 mM, 0.8 mM, or 1.0 mM for brain glucose; 25 or 40 for LPR; etc.)*

94.3% (33/35) — SA: 23, A: 10, N: 1, D: 1, SD: 0, N/A: 0

Sub-question: *22.R2. depth of CMD catheter tip*

82.9% (29/35) — SA: 19, A: 10, N: 5, D: 1, SD: 0, N/A: 0

Sub-question: *23.R2. duration of adequate CMD system (i.e., machine, catheter, etc.) functioning and total duration of CMD monitoring*

94.3% (33/35) — SA: 21, A: 12, N: 2, D: 0, SD: 0, N/A: 0

Sub-question: *24.R2. if available, hourly plasma glucose records in addition to cerebral glucose records*

74.3% (26/35) — SA: 16, A: 10, N: 8, D: 1, SD: 0, N/A: 0

Sub-question: *25.R2. if intravenous glucose supplementation is provided, the rate (mmol/hour) and concentration of the supplementation alongside the set of, if available, associated plasma and cerebral glucose records*

88.6% (31/35) — SA: 14, A: 17, N: 3, D: 1, SD: 0, N/A: 0

Question 26.R1

Statement: *Although center-dependent, the lack of automated streaming of CMD data to electronic medical records poses a barrier to the efficient clinical implementation of CMD monitoring.*

97.1% (34/35) — SA: 18, A: 16, N: 0, D: 0, SD: 1, N/A: 0

Question 27.R2

Statement: *Although region and center-dependent, lack of approval of CMD for routine clinical use by some regulatory agencies presents a barrier to the clinical implementation of CMD monitoring.*

91.4% (32/35) — SA: 17, A: 15, N: 3, D: 0, SD: 0, N/A: 0

Round 3 results

34 panelists participated in this round.

Question 1.R1

Main Statement Stem: *In a prioritized approach to treating deranged metabolism with the intention of lowering the LPR, the following general sequence of priorities is suggested, while acknowledging that these parameters are often managed simultaneously depending on the clinical context:*

Sub-question: *1. ICP should be addressed with priority (target ≤ 20 mmHg),*

94.1% (32/34) — SA: 25, A: 7, N: 1, D: 0, SD: 1, N/A: 0

Sub-question: *2. then PbtO2 (target ≥ 15 mmHg),*

94.1% (32/34) — SA: 15, A: 17, N: 1, D: 0, SD: 1, N/A: 0

Sub-question: *3. and finally, brain glucose should be monitored, with investigation and management of systemic causes for levels below 1.0 mM.*

94.1% (32/34) — SA: 14, A: 18, N: 1, D: 0, SD: 1, N/A: 0

Sub-question: *4. Further interventions (e.g., targeting CPP) should be considered for refractory metabolic derangement as indicated by a persistently increased LPR (> 25).*

79.4% (27/34) — SA: 10, A: 17, N: 5, D: 1, SD: 1, N/A: 0

Question 2.R1

Statement: *Worsening CMD values may indicate tissue vulnerability and can help inform the decision to defer, modify, or abort a neurological wake-up test or a trial of sedation lightening.*

88.2% (30/34) — SA: 12, A: 18, N: 2, D: 1, SD: 1, N/A: 0

Question 3.R1

Statement: *In TBI patients with a CMD pattern suggestive of ischemia (e.g., LPR > 25 and pyruvate < 70 μM), the initial response should be to evaluate and optimize cerebral perfusion pressure, brain tissue oxygenation, cerebral autoregulation, and brain glucose levels. If the ischemic pattern persists despite these measures, a short (e.g., up to 2 hours) trial of normobaric hyperoxia may be considered as a diagnostic challenge to assess the responsiveness of the metabolic derangement.*

76.5% (26/34) — SA: 2, A: 24, N: 5, D: 2, SD: 1, N/A: 0

Question 4.R2

Statement: *The administration of alternative energy substrates, such as lactate or ketone bodies, to manage neuroglycopenia is an investigational strategy that requires further study before it can be recommended for clinical use.*

87.9% (29/33) — SA: 18, A: 11, N: 4, D: 0, SD: 0, N/A: 1

Question 5.R2

Statement: *Systemic (plasma) glucose levels should be considered when interpreting cerebral microdialysis glucose data.*

97.1% (33/34) — SA: 22, A: 11, N: 1, D: 0, SD: 0, N/A: 0

Question 6.R2

Statement: *Elevated cerebral glycerol is an indicator of cell death and damage from secondary injury after traumatic brain injury (TBI).*

85.3% (29/34) — SA: 6, A: 23, N: 1, D: 4, SD: 0, N/A: 0

Question 7.R2

Statement: *An increase in cerebral glutamate is indicative of secondary injury, such as from excitotoxicity, after traumatic brain injury (TBI).*

79.4% (27/34) — SA: 8, A: 19, N: 7, D: 0, SD: 0, N/A: 0

Question 8.R2

Statement: *Increased cerebral glutamate is associated with poor clinical outcome.*

73.5% (25/34) — SA: 5, A: 20, N: 7, D: 2, SD: 0, N/A: 0

Question 9.R1

Statement: *In patients undergoing ICH resection, CMD may be considered as an adjunct to standard monitoring to assess the metabolic response to surgery. With the catheter tip placed in the perihematomal zone, worsening or non-recovering metabolic derangements despite resection may signal an evolving secondary injury, prompting further investigation to identify potential causes.*

60.6% (20/33) — SA: 6, A: 14, N: 11, D: 2, SD: 0, N/A: 1

Question 10.R2

Statement: *If available, plasma glucose records should be considered core reporting items in conjunction with cerebral glucose records.*

94.1% (32/34) — SA: 19, A: 13, N: 2, D: 0, SD: 0, N/A: 0
